# Tumor-Associated Extracellular Matrix: How to Be a Potential Aide to Anti-tumor Immunotherapy?

**DOI:** 10.3389/fcell.2021.739161

**Published:** 2021-10-18

**Authors:** Yingying He, Tao Liu, Shuang Dai, Zihan Xu, Li Wang, Feng Luo

**Affiliations:** ^1^Department of Medical Oncology, Lung Cancer Center, West China Hospital, Sichuan University, Chengdu, China; ^2^Oncology Department, People’s Hospital of Deyang City, Deyang, China; ^3^Department of Oncology, The First Affiliated Hospital of Chengdu Medical College, Chengdu Medical College, Chengdu, China

**Keywords:** extracellular matrix, cancer-immunity cycle, T-cell lymphocyte, immunotherapy, proteoglycans

## Abstract

The development of cancer immunotherapy, particularly immune checkpoint blockade therapy, has made major breakthroughs in the therapy of cancers. However, less than one-third of the cancer patients obtain significant and long-lasting therapeutic effects by cancer immunotherapy. Over the past few decades, cancer-related inflammations have been gradually more familiar to us. It’s known that chronic inflammation in tumor microenvironment (TME) plays a predominant role in tumor immunosuppression. Tumor-associated extracellular matrix (ECM), as a core member of TME, has been a research hotspot recently. A growing number of studies indicate that tumor-associated ECM is one of the major obstacles to realizing more successful cases of cancer immunotherapy. In this review, we discussed the potential application of tumor-associated ECM in the cancer immunity and its aide potentialities to anti-tumor immunotherapy.

## Introduction

The adaptative immune response protects against tumors (Ribas, 2015). However, neoplastic cells have developed strategies to avoid immune detection and elimination, that is considered as a hallmark of cancer (Hanahan and Weinberg, 2011). The therapeutic potential of host-versus-tumor effect can be motivated with novel immune therapies, including immune checkpoint inhibitors (ICIs), adoptive cell therapy, and vaccines (Baxevanis et al., 2009). CD4^+^ and CD8^+^ T lymphocytes comprise primary effector cells against tumors. Any step from the release of cancer cell antigens to the killing of cancer cells is regulated by several regulatory mechanisms. Immune checkpoints are inhibitory regulators that act as “breaks” on the immune response, which can be targeted by ICIs (Zhang and Zheng, 2020). Although a variety of ICIs have been significantly successful in cancer treatment, the curative effect remains poor (Li et al., 2019).

It is an urgent issue to solve the intrinsic and secondary resistance of ICIs. Herein, we have to mention ECM, a non-cellular three-dimensional macromolecular network composed of collagens, proteoglycans (PGs)/glycosaminoglycans (GAGs), elastin, fibronectin (FN), laminins, and several other glycoproteins (Theocharis et al., 2016). Whether in normal tissue or tumors, matrix components and cell adhesion receptors combine with each other to form a complex network where diverse cells reside. For many years, ECM was regarded as an inert cellular scaffold, which only provided structure for the cells. In the past two decades, people have discovered more functions that affect both biochemical and biophysical processes in cells, and ECM has been regarded as a reservoir and binding site of bioactive molecules (Brown, 2011). Cell surface receptors transmit signals from ECM to cells to regulate a variety of cell functions, such as survival, growth, migration, differentiation and immunity, which are essential for maintaining normal homeostasis (Humphrey et al., 2014).

Essentially, ECM is a highly dynamic entity that continuously undergoes remodeling mediated by several matrix-degrading mechanisms during normal and pathological conditions (Lu et al., 2012; Yue, 2014). Continuous modification or dysregulation of ECM can lead to tumorigenesis (Walker et al., 2018). As far as we know, solid tumors characterized by disrupted tissue homeostasis and loss of differentiation phenotype are always accompanied by alterations in the composition, tissue and mechanical properties of the ECM (Eble and Niland, 2019). A large number of studies have shown that tumor-associated ECM is involved in promoting the growth, invasion, metastasis, and angiogenesis of tumor cells (Watanabe, 2010; He et al., 2016; Erdogan and Webb, 2017; Najafi et al., 2019), but it resists cell death and drug diffusion (Mason et al., 2017; Hwang et al., 2019).

**FIGURE 1:**
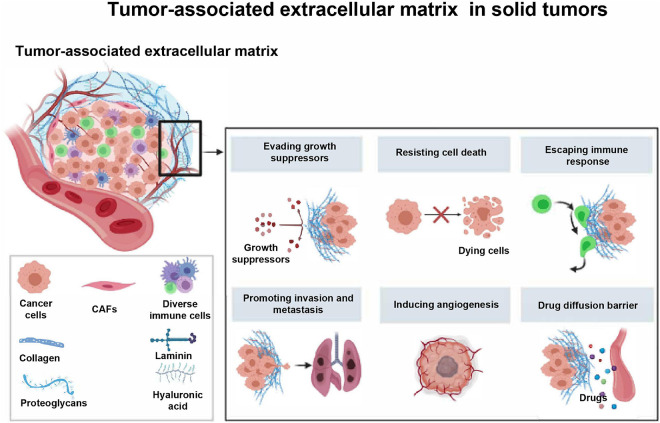
The major components and roles of tumor-associated extracellular matrix in solid tumors. The tumor-associated ECM has been considered as a newly formed network that is essential for the protection of tumor cells and promotion of tumor progression. CAFs, cancer associated fibroblasts.

In addition, a recent study showed that the remodeling of neoplastic ECM will regulate the immune system (Mohan et al., 2020), which is usually regarded as an obstacle to immune response (Vaday and Lider, 2000; Figure 1). Simply put, immune cells are actively involved in regenerating damaged tissues and promoting the deposition and formation of ECM (Pickup et al., 2014). In turn, the tumor-associated ECM contributes to the development of an immunosuppressive network, where cancer cells intertwine with fibroblasts, immune cells, endothelial cells, and other sorts of stromal cells. Within the newly formed network, secreted cytokines and chemokines can lead to immune escape of tumor (Nikitovic et al., 2014; Chakravarthy et al., 2018; Gao et al., 2019).

Up to now, the clinical trial data of cancer immunotherapy have confirmed that less than one third of the patients have a lasting and significant therapeutic effect (Wang et al., 2020). One of the predictors of therapeutic effect is tumor-infiltrating T cells, which are closely related to immune hot tumors or immune cold tumors. The hot tumors characterized by molecular markers of T cell infiltration and immune activation show a high response rate to immunotherapy such as anti-programmed death ligand 1 (PD-L1)/PD-1 therapy, whereas, cold tumors show striking features of T cell absence or exclusion (Gajewski, 2015; Zemek et al., 2019). Emerging evidence suggests that a common rate-limiting step is the immunostat function. Components in ECM and its proteolytic remodeling products can regulate the immune response, and act as an immune rheostat or “immunostat” (Pao et al., 2018). How does ECM affect the anti-tumor immunity? Will ECM be closely related to immune cold tumors? In view of these two issues, we will focus on the relationship between ECM and tumor immune response, and critically review the role of ECM in cancer immunity and its potential combination with cancer immunotherapy.

## Extracellular Matrix in Cancer

Extracellular matrix is an intricate network of extracellular-secreted macromolecules, such as collagens, enzymes and glycoproteins, with main functions dealing with structural scaffolding and biochemical support of cells and tissues (Lu et al., 2012). Generally, ECM can be divided into the basement membrane (BM) and the interstitial matrix (IM), to support epithelial/endothelial cell behavior and support the underlying stromal compartment and peri-cellular membrane, respectively (Theocharis et al., 2016). Degradation of the surrounding ECM is an essential part of the growth of invasive cancer, and it’s also the main cause of destroying normal tissue (Madsen and Bugge, 2015). Most importantly, the degradation of ECM is accompanied by the deposition of a different tumor-specific ECM (Schedin and Keely, 2011), which usually increases in density and stiffness (Cox and Erler, 2014).

### Basement Membrane

Basement membrane, composed of collagens, laminins, PGs, and FN (Haraida et al., 1996), sites at the interface between parenchyma and connective tissue, providing an anchoring sheet-like layer for parenchymal cells in order to be held together preventing them from being torn (Halfter et al., 2015). In epithelial cancers, BM acts as a structural barrier of invasion, intravasation and extravasation of cancer cells. During the development and progression of cancer, changes in BM are often observed. Generally, cancer cells invade the BM through such means as producing ECM remodeling enzymes (e.g., Matrix Metalloproteinases), using natural pores in BM, or forcing their way through those pores (Chang and Chaudhuri, 2019). In addition, a more complicated phenomenon is observed, that is, invasion also depends on the contact of cancer cells with collagen fibers in the underlying stroma (Nissen et al., 2019). In areas assembled by sheet-like arrangements of laminins (Gordon-Weeks et al., 2019), FN (Lin et al., 2019) as well as collagens I, III, and IV, the BM was excessively thickened (Nissen et al., 2019). This structural arrangement effectively divides the tumor into cancer cells nests and stromal regions, thereby producing the malignant behavior of cancer cells (Eble and Niland, 2019) and regulating tumor immune response (Vaday and Lider, 2000).

### Interstitial Matrix

Under physiological condition, IM is a loose ECM that goes deep into the BM, which is composed of collagens I and III, elastin fibers and glycoproteins. Fibroblasts, resident immune cells, vasculature, and lymphatics are all embedded within it (Bruckner-Tuderman et al., 2010; Mecham, 2012). However, in some tumors, collagen fibers in IM are thicker, more organized and denser due to the increased deposition of collagen fibers (Drifka et al., 2016b; Nissen et al., 2019). With the development of cancers, stromal collagen fibers become increasingly aligned, particularly at the edge of cancers, thus promoting the invasion of cancer cell (Conklin et al., 2011; Burke et al., 2013; Han et al., 2016). Furthermore, lysyl oxidase (LOX) family catalyzes the formation of collagen cross-linking (Laczko and Csiszar, 2020). In tumors, the increased expression of LOX leads to excessive cross-linking of collagen, and at the same time, collagen deposition increases, increasing the stiffness, and leading to solid stress in the tumor (Choi et al., 2017). Besides the change of collagen, various glycoproteins in the ECM are also expressed pathologically, such as versican (VACN) and FN. All of them form a niche to facilitate cells migration, adherence and metastasis (Theocharis et al., 2016).

### Major Components of Extracellular Matrix

#### Collagens

Collagen is one of the main components of ECM, which participates in cancer fibrosis and structural formation of solid cancers together with matrix glycoproteins, such as FN, laminins, elastin, and versican (Nissen et al., 2019). The biosynthesis of collagen is always regulated by fibroblasts, cancer cells and other stromal cells (e.g., macrophages) through mutated genes, transcription factors, multiple signaling pathways, and receptors (Sorushanova et al., 2019). Other substances in ECM, such as FN, hyaluronic acid (HA), laminin, and matrix metalloproteinases (MMPs), interact with collagen through integrins, discoidin domain receptors (DDR), tyrosine kinase receptors, and some signaling pathways to influence the behavior and activity of cancer cells. Changes of the internal matrix composition of cancer eventually form a mutual feedback loop, which affects the prognosis, recurrence and treatment resistance of cancer (Xu et al., 2019).

Several advanced stages of cancer, such as pancreatic cancer (Vennin et al., 2015), breast cancer (Nolan et al., 2020), colon cancer (Tabuso et al., 2021), are characterized by a desmoplastic reaction, which is manifested by extensive deposition of fibrillar collagens in ECM, presenting thicker, more organized and more highly packed collagen fibers. Deposition of these fibers leads to typical stiff matrix of cancer (Stylianopoulos et al., 2018), especially in the tumor regions nearby the BM, where increased fiber density can enhance the invasion of cancer cell (Jurmeister et al., 2020) and cause the poor prognosis of several cancers such as breast cancer (Bredfeldt et al., 2014), pancreatic cancer (Drifka et al., 2016a), gastric cancer (Ohno et al., 2002), and oral squamous cell carcinomas (Li et al., 2013).

Solid stress results from a combination of rapid proliferation of cancer cells, cell-ECM interactions and massive ECM deposition, all of which interact with resistive forces from the surrounding normal tissue (Löffek et al., 2016). Cancer cells sense these mechanical changes of the ECM through specialized transmembrane receptors including integrins (Cooper and Giancotti, 2019), DDRs (Coelho and McCulloch, 2018) and syndecans (Kang et al., 2018), and transform them into biological reactions to regulate cell functions.

Integrins are the main receptors of collagen, which are widely expressed and promote cell migration, and may be a key pathway for tumor angiogenesis, chemotherapy resistance, and metastasis (Naci et al., 2015; Zeltz and Gullberg, 2016; Wu et al., 2019). Integrin α11β1, a stromal cell-specific receptor for fibrillar collagens, is overexpressed in carcinoma-associated fibroblasts (CAFs). Non-small cell lung carcinoma (NSCLC) xenografts in α11 knockout (α11^–/–^) severe combined immune deficient (SCID) mice was significantly impeded, as compared with wild-type (α11^+/+^) SCID mice, which showed that collagen cross-linking was associated with stromal α11 expression, and the loss of tumor-stromal α11 expression was related to collagen reorganization and the decrease of stiffness (Navab et al., 2016). The forced expression of β1 integrin significantly stimulated Src and extracellular signal-regulated kinase (ERK) phosphorylation, increased cell stiffness, and accelerated cell motility, suggesting that the integrin signaling pathway activated in a tumor environment with collagen deposition is responsible for low cell elasticity and high metastatic ability (Chen et al., 2014). Integrins also control local activation of transforming growth factor β (TGF-β) in ECM and cell-surface reservoirs. Integrin-dependent activation of TGF-β has become an important mechanism, through which tissue-borne cells guide circulating and resident immune cells (Nolte and Margadant, 2020). Therefore, specific inhibition of integrin-mediated cancer cell-collagen interaction may provide an opportunity for therapeutic intervention in the metastasis and spread of certain cancers like ovarian cancer (Huang et al., 2020).

In addition, DDRs (DDR1 and DDR2), non-integrin collagen receptors, are expressed on the surface of tumor cell, belonging to the receptor tyrosine kinase (RTK) family. DDRs show delayed and sustained activation when interacting with collagen (Orgel and Madhurapantula, 2019), and DDR-collagen signaling plays an important role in cancer proliferation and progression (Gadiya and Chakraborty, 2018). When DDR2 is activated, it will trigger a signaling cascade involving protein tyrosine phosphatase-2 (SHP-2), SRC, and MAP (mitogen-activated protein) kinases with SH2 domain. A study showed that knockdown of DDR2 in the NA13 cell line, B16F10 cell line and mammary tumor cell line E0771 could enhance the sensitivity of tumor cells to anti-PD-1 therapy *in vitro*. *In vivo*, tumor load reduction and higher CD8^+^T cells infiltration in tumor were observed in tumor-bearing mice treated by dasatinib (a tyrosine kinase inhibitor of DDR2) plus anti-PD-1 (Tu et al., 2019). A clinical trial (NCT01643278) showed that dasatinib and ipilimumab can be safely administered to patients with gastrointestinal stromal tumor (GIST) and sarcoma. However, dasatinib was not synergistic with ipilimumab, as there was limited clinical efficacy with the combination (D’Angelo et al., 2017).

In addition to the above-mentioned effects of collagen, the relationship between collagen and tumor-associated macrophages (TAMs) in anti-tumor immunity should not be underestimated. TAMs, regarded as a major limitation for the efficacy of cancer immunotherapy, always suppress the activity of tumor-infiltrating T cells to support tumor growth (Ngambenjawong et al., 2017). Co-culture assays with primary T cells showed that macrophages cultured in high-density collagen had low efficiency in attracting cytotoxic T cells, and were capable of inhibiting T cell proliferation, which indicated that a high collagen density can guide macrophages to acquire an immunosuppressive phenotype (Larsen et al., 2020).

#### Proteoglycans

Proteoglycans, as components of ECM, play a key role in providing intrinsic signals needed to coordinate key events of regulating cancer immunity (Schaefer et al., 2017). PGs are complex molecules consisting of a protein core into which one or more glycosaminoglycan (GAG) chains are covalently linked (Piperigkou et al., 2016). The bound GAGs can be the heparan sulfate (HS), the chondroitin sulfate/dermatan sulfate (CS/DS) or the keratan sulfate (KS) (Iozzo and Schaefer, 2015). PGs are highly implicated in the processes of cancer-associated inflammation and regulate key events, respectively, to both innate and adaptive immunity (Schaefer et al., 2017). In this section, we will list three members of PGs that have been deeply studied. Other members may appear in other sections.

Versican, a member of the hyalectan family of large chondroitin sulfate PGs (CSPGs), has been proved to be overexpressed in many cancers (Papadas et al., 2020). VCAN can act as a tissue “landing strips” for inflammatory cells from the circulation via binding tumor necrosis factor stimulated gene-6 (TSG-6) and inter alpha trypsin inhibitor (IαI) (Wight et al., 2014). In addition, VCAN interacts with inflammatory cells either indirectly via HA or directly via receptors such as CD44, P-selectin glycoprotein ligand-1 (PSGL-1) and toll-like receptors (TLRs), and activates signaling pathways to promote the synthesis and secretion of inflammatory cytokines such as tumor necrosis factor alpha (TNFα), interleukin 6 (IL-6), and nuclear factor kappa B (NFκB) (Kim et al., 2009; Wight et al., 2014).

Biglycan (BGN), a member of small leucine rich PGs (SLRPGs) (Schaefer et al., 2017), is overexpressed and secreted by various cancers (Wang et al., 2011; Liu et al., 2014; Fujiwara-Tani et al., 2020), which is related to the regulation of immunological responses (Neill et al., 2015). However, the oncogenic or tumor suppressive potential of BGN remains unclear. Compared to BGN^low/neg^ HER-2/neu^+^ fibroblasts, BGN^high^ HER-2/neu^+^ fibroblasts were less tumorigenic and had increased immunogenicity in immune competent mice, possibly due to upregulation of major histocompatibility complex (MHC) class I surface antigens and reduced expression levels of TGF-β isoforms and TGF-β receptor 1 (Subbarayan et al., 2018). Soluble BGN activates the primary reaction of adaptor molecule myeloid differentiation 88 (MyD88), recruits neutrophils and macrophages by using the Toll like receptor 2/4 (TLR2/4) signaling pathways, or activates the domain-containing adaptor of Toll/interleukin 1R (IL-1R), and induces the activity of interferon β (IFN-β) recruited by T-lymphocyte (Zeng-Brouwers et al., 2014).

In addition to VCAN and BGN, HS proteoglycans (HSPGs) play a multifunctional role in inflammation, such as modulating multiple steps in the cascade of leukocyte recruitment (Kumar et al., 2015), activating lymphocytes (Wrenshall et al., 1994), and inducing phenotypic maturation of murine immature dendritic cells (DCs) with upregulation of I-A, CD40, CD54, CD80, and CD86 (Kodaira et al., 2000). El Ghazal et al. (2016) found that targeting of DC glycan sulfation through mutation in the heparan sulfate biosynthetic enzyme N-deacetylase/N-sulfotransferase-1 (Ndst1) in mice (Ndst1^f/f^ LysMCre^+^) increased DC maturation and inhibited trafficking of DCs to draining lymph nodes, and Lewis lung carcinoma tumors of Ndst1^f/f^ LysMCre^+^ mice were reduced in size (El Ghazal et al., 2016).

#### Glycosaminoglycans

Hyaluronic acid is a simple linear and unsulfated GAG composed of repeating units of N-acetylglucosamine (GlcNAc) and glucuronic acid (GlcUA), which is accumulated in a variety of human solid tumors (Nikitovic et al., 2015; Caon et al., 2020). The biological activities of HA depend on its molecular weight and the receptors interacting with it (Monslow et al., 2015). HA is initially synthesized as high molecular weight (HMW) polymers beyond 500 kDa. These HMW-HA functions are mediated by constitutively expressed receptors, including CD44, lymphatic endothelial receptor (LYVE-1) and HA receptor for endocytosis (HARE) (Du et al., 2013) to maintain homeostasis and restrain cell proliferation and migration in normal tissues (Mueller et al., 2010; Tian et al., 2013). HMW-HA can be fragmented into low molecular weight (LMW) polymers between 7 and 200 kDa by hyaluronidases and free radicals (D’Agostino et al., 2015). These LMW-HA can promote inflammation, immune cell recruitment and epithelial cell migration (Törrönen et al., 2014; Litwiniuk et al., 2016). These functions are mediated by receptors for HA-mediated motility (RHAMM) and TLR2/4, which coordinate signaling with CD44 and other HA receptors (Misra et al., 2015). Elevated HMW-HA production in the absence of fragmentation is linked to cancer resistance, however, tumor cells always promote HA fragmentation associated with driving and maintaining malignant progression (Liu M. et al., 2019). For instance, fragmentated HA activates RhoGTPase signaling, rapamycin (mTOR) pathway and CDC42 signaling by binding CD44 to maintain proliferative signaling (Mattheolabakis et al., 2015; Skandalis et al., 2019).

The components mentioned above are only a small population of thousands of ECM components. They not only perform their respective functions, but also interact with each other and participate in the dynamic changes of ECM and immune response. In this article, we have discussed certain aspects in, such as how they affect the cancer-immunity cycle.

## The Cancer-Immunity Cycle and Anti-Tumor Immunotherapy

According to the theory of immune surveillance, cells and tissues are constantly under the control of the immune system (Ribatti, 2017). Cancer is a systemic disease, with prolonged inflammation as a hallmark (Singh et al., 2019). T immune system serves as a hindrance to cancer formation and progression, actively participating in resisting or eradicating the formation and progression of incipient cancer, advanced tumors, and micro-metastases (Abbott and Ustoyev, 2019). However, both extrinsic and intrinsic cancer inflammations can result in immunosuppression, thereby providing a preferred background for tumor development (Nakamura and Smyth, 2017).

**FIGURE 2:**
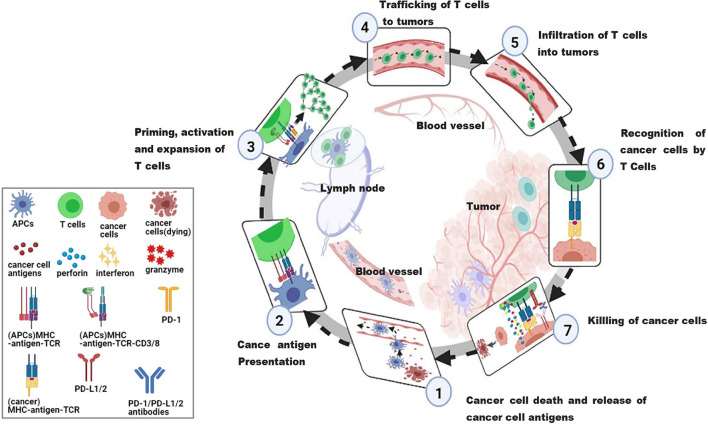
The cancer-immunity cycle. An effective anticancer immune response is a cyclic process, theoretically, which can be divided into seven major steps, starting with the release of antigens from the cancer cell and ending with the killing of cancer cells. Importantly, these steps must be repeated to amplify and broaden T cell responses. Each step is described as above, with the primary cell types involved and the anatomic location of the activity listed. APCs, antigen presenting cells, a type of cell in the body that takes up, processes and transmits antigenic information, and induces an immune response from T and B cells; MHC, major histocompatibility complex, involved in antigen presentation and the associated immune response, divided into MHC I and MHC II; MHC I, responsible for presenting viral, tumor or parasitic antigens; MHC II, responsible for initiating the immune response in bacterial infections; TCR, T cell receptor, an antigen receptor on the surface of T cells that recognizes pMHC complex; PD-1, programmed cell death protein 1, located on the surface of T cells and primary B cells; PD-L1/2, programmed cell death 1 ligand 1/2, ligand for PD-1.

Anti-tumor immunotherapy aims to combine with the host immune system to initiate or reinitialize a self-sustaining cycle of cancer immunity, so as to generate effective anti-tumor immune responses. In order to effectively kill cancer cells, it is necessary to initiate and repeat several stepwise events (Chen and Mellman, 2017). The steps are summarized as the cancer-immune cycle (Figure 2). In summary, there are 7 steps. Step 1, the antigens of tumorigenic cancer cell are released and captured by antigen-presenting cells (APCs). Step 2, the captured antigens are presented to T cells on MHC I and MHC II molecules by APCs. Step 3, the response of effector T cells (T_EFF_) against the cancer-specific antigens is initiated and activated, and then T_EFF_ is amplified. Step 4, the activated T_EFF_ migrate toward tumors. Step 5, the activated T_EFF_ arrive at lesions and infiltrate the tumor bed. Step 6, activated T_EFF_ specifically recognize and bind cancer cells through the interactions between T cell receptor (TCR) and their homologous antigens bound to MHC I. Step 7, these activated T_EFF_ finally kill their target cancer cells through releasing perforin, granzyme, and interferon-gamma (IFN-γ). In this step, additional cancer cell antigens are released, which can restart step 1 again and strength the response in continuous rotation of the cycle (Chen and Mellman, 2013). However, negative feedback exists in cancer-immunity cycle, such as immune checkpoints (Kong, 2020), arginase (Caldwell et al., 2018) and vascular endothelial growth factor (VEGF) (Fukumura et al., 2018), preventing amplification of continued immune signals, and inhibiting or arresting the anticancer immune response. Several immune escape mechanisms have been described in many studies, including the lack of tumor-antigen recognition (Salzer et al., 2020), the resistance to cell death (Galluzzi et al., 2018), and the production of immunosuppressive molecules by tumor cells (Jie et al., 2013).

Unfortunately, only a few cancer patients can do very well in the cancer-immunity cycle, and a significant fraction of patients manifests innate or acquired resistance to those therapies (Galluzzi et al., 2018). A better understanding of resistance mechanisms depicts a central goal to avoid or overcome immunotherapy resistance, thereby improving the outcome of cancer patients. TME is a mechanism of resistance other than tumor cells themselves (Darragh et al., 2018; Horvath et al., 2020). ECM is the non-cellular stroma of TME, which hosts tumor cells, endothelial cells, mesenchymal cells, and various immune cells (Arneth, 2019). Growing research has shown that the remodeling of ECM plays an important role in shaping the inflammation and immune milieu of the tumor. The remodeling of ECM cytoskeleton, structural plasticity and mechanical forces are crucial factors for the transportation, activation, and formation of immunological synapses (Huse, 2017).

## Multiple Roles of Extracellular Matrix in Regulating the Cancer-Immunity Cycle

As mentioned above, each step of the Cancer-Immunity Cycle requires the coordination of numerous factors, both stimulatory and inhibitory in nature (Chen and Mellman, 2017). According to current research results, ECM is able to participate in the regulation of multiple steps in the Cancer-immune Cycle, however, the results are scattered and not generalized. In order to have a more systematic and comprehensive understanding of the impacts of ECM on the cancer-immunity cycle, we will summarize them in the order of the steps of this cycle.

### Stiff Extracellular Matrix Inhibits Cancer Cell Death and Decreases Release of Cancer Cell Antigens

Extracellular matrix in tumors is approximately at least 1.5-fold stiffer than that in the surrounding normal tissue (Najafi et al., 2019). By applying physical forces through the stiffened ECM on host tissues, tumors can enhance cell-ECM adhesion and break cell-cell contacts, leading to its growth (Dupont, 2016; Gkretsi and Stylianopoulos, 2018) and survival (Piersma et al., 2020). Inducing collagen cross-linking in a stiff ECM can enhance phosphoinositide 3-kinase (PI3K) activity, which improves the viability of cancer cells (Gao et al., 2020). Survival of cancer cells is also affected by the release of metalloproteinases (MMPs) from cells (Grzelczyk et al., 2016). MMPs, a family of proteolytic enzymes, degrade multiple components of ECM (Winer et al., 2018) and interact with integrins, thus promoting the survival of intracellular signal transducer and activator of transcription 3 (STAT3) (Najafi et al., 2019). HA activates RhoGTPase signaling, mTOR pathway and CDC42 signaling by binding CD44 to maintain proliferative signaling (Mattheolabakis et al., 2015; Skandalis et al., 2019). Moreover, ECM stiffness is indirectly involved in activation of ERK pathway, which contributes to the proliferation of cancer cells (Paszek et al., 2005).

As we all know, chemotherapeutic agents are always used to endow tumor cells with immunogenicity and/or increase their immunogenicity by inducing the death of immunogenic cell, thus increasing the release of cancer cell antigens (Garg et al., 2017; Wang et al., 2018). However, in addition to promoting survival of cancer cells by ECM, the stiffness of ECM is an obstacle to the effective uptake or delivery of drugs to the intratumoral area (Kaylan et al., 2016). In short, the enhancement of survival potential of tumor cells leads to their freedom from growth inhibition, which also reduces cell death and the release of cancer cell antigens (Gubin et al., 2015). As the first and key step for initiating anticancer immunity (Kroemer et al., 2013), a reduction in cancer cell antigen release will cripple the Cancer-Immunity Cycle.

### Extracellular Matrix Interferes With Cancer Antigen Presentation

The functional basis for ICIs response is the immunogenicity of a tumor, which is mainly determined by tumor antigenicity and the efficiency of antigen presentation (Wang et al., 2019). APCs, including macrophages, DCs and B cells, are responsible for presenting antigens and triggering immunity by different mechanisms (Blander et al., 2017). DCs are the sentinel APCs of the immune system and act as initiators of antigen-specific T cell responses (Constantino et al., 2017). Nevertheless, only mature DCs are able to induce anti-tumoral immunity, while antigens presented by immature DCs may lead to immune tolerance and fail to induce a T-cell response (Wculek et al., 2020).

It’s worth noting that the ultimate fate of DC functions is determined by signals from the microenvironment (Leenman et al., 2010). ECM components might induce a DC phenotype with low immunogenicity (Sprague et al., 2011). Studies on the angiogenic and immune profile of murine myeloid DCs upon interaction with laminins (a family of large molecular weight glycoproteins) environments have shown that murine ovarian tumors produce several types of laminins and that DCs cultured on those laminins upregulate both AKT and MEK signaling pathways and decrease the immunological capacities, which contributes to their complicity in tumor growth (Aumailley, 2013; Phillippi et al., 2020). As mentioned in the second section, some matrix components, such as HS and HA, have immunoregulatory properties in experimental models. In mice, targeting the sulfation of HS on DCs through mutation in the heparan sulfate biosynthetic enzyme N-deacetylase/N-sulfotransferase-1 (Ndst1) can endow DCs with increased maturation and reduced chemokine-dependent trafficking to draining lymph nodes (El Ghazal et al., 2016), where mature DCs present the peptide to naive T cells in a complex with MHC proteins to initiate downstream reactions (Jakubzick et al., 2017).

Besides HS, HA can also regulate the maturation of DCs in a TLR4 dependent manner (Termeer et al., 2002). It was found that the exposure of DCs to HA fragments increased the expression of activation markers as MHC II, CD80, CD86, and CD40, and promoted DCs activation (Alaniz et al., 2009). When activated, DCs migrate to the draining lymph nodes and present the antigen to naive T cells (Worbs et al., 2017). Furthermore, HA can form provisional matrices with VACN. The HA-VACN interaction is important for the recruitment of inflammatory cells including neutrophils, macrophages and T cells (Andersson-Sjoland et al., 2015; Wight, 2017). The interaction between FN (a large glycoprotein of ECM) and integrin β1 in macrophages can promote TLR 2/4 signaling pathways to enhance expression of pro-inflammatory mediators and phagocytosis by macrophages (Fei et al., 2018). When located near the proximity of dying cancer cell, DCs together with macrophages can mobilize to lymphoid organs and present tumor antigens to adaptative immune cells, thus enabling the resolution of the tumor (Gardner and Ruffell, 2016).

### Extracellular Matrix Influences the Initiation and Activation of Effector T Cells

Generally, naive T cells are located within lymph nodes to encounter with antigen-loaded DCs and be activated (Groom, 2015). In lymphoid nodes (LNs), various stromal cell subsets create dense 3D cellular networks, which provides the opportunity for naive T cells to interact with antigens presented by DCs, resulting in the initiation of an immune response (Mempel et al., 2004). The ultimate fate of T cell in LNs is determined by interactions with the matrix framework and various other immune components (Bousso and Robey, 2003). Fibroblastic reticular cells (FRCs), lymphatic endothelial cells (LECs), and blood endothelial cells (BECs) are the main LN stromal cells (SCs) (Fletcher et al., 2015). FRCs provide the three-dimensional scaffold in the LN niche, and are characterized by expressing podoplanin (gp38) and ECM proteins, such as laminins and collagens (Martinez et al., 2019). In the LN paracortex, collagen fibers, laminins and FRCs make up microchannels known as the conduit network. The conduit center has abundant collagen type I; the BM is rich with laminin α4β1γ1 (411), laminin α5β1γ1 (511), collagen type IV, and FN (Katakai et al., 2004). Bridging the subcapsular sinus and high endothelial venules (HEVs) by the conduit allows small molecules less than 70 kilodaltons, such as soluble antigens, chemokines and cytokines, to be delivered into the paracortex (Gretz et al., 2000; Roozendaal et al., 2008).

In LNs, immunity or tolerance induction can affect the expression of laminin α4 and α5 in all SCs (Warren et al., 2014). In response to immunity and inflammation, laminin α5 is upregulated (Li et al., 2020). On the contrary, α4 increases for tolerance induction. Functionally, laminin 411 and laminin 511 serve as co-inhibitory and co-stimulatory ligands for CD4^+^ T cells, respectively. Laminin 411 inhibits the activation of CD4^+^T cells and the polarization of Th1, Th2, and Th17, but facilitates the induction of polarization of T regulatory cells (iTreg). Laminin 511 is recognized by CD4^+^ T cells through α6 integrin and α-dystroglycan to inhibit T cells activation, proliferation and differentiation (Simon et al., 2019).

T cells are initiated and activated in the tumor draining lymph nodes (TDLNs), and obtain their effector functions to migrate toward the tumor (Rotman et al., 2019). Interactions of T cells with major ECM structural components within TDLNs can regulate the function of T cells. In the T cell zone, FRCs support naive CD4^+^ and CD8^+^ T cell or memory precursor effector cell homeostasis by producing IL-7, IL-15, and CCL19. Moreover, the presentation of antigens linked to a stiff surface has been demonstrated to impair TCR-mediated T cell activation, suggesting that TCR is affected by mechanical force (O’Connor et al., 2012; Saitakis et al., 2017; Zhang et al., 2021).

### Extracellular Matrix Regulates Immigration of T Cells, Including Trafficking and Infiltration to Tumors

Effector T cells from LNs to the tumor site are critical for the density and diversity of tumor-infiltrating T cells, which is closely related to the prognosis and curative effect of cancer immunotherapies (Bindea et al., 2013). This view is accepted as immune hot/cold tumors (Bonaventura et al., 2019). The process of T-cell trafficking is highly dynamic and governed by a complex array of mechanisms, involving complex interactions between T cells and endothelial cells (ECs) (Vaday and Lider, 2000; Vaday et al., 2001). In brief, during this process, T cells need toattach to the endothelium for a short time at first, then roll, firmly adhere and are activated on the endothelial surface, and finally extravasate through the blood vessel wall to tumor site (Masopust and Schenkel, 2013; Chimen et al., 2017).

T-cell trafficking is also highly dependent on microenvironmental clues. T cells utilize the porous three-dimensional ECM as a scaffold for both integrin-dependent and receptor-independent amoeboid motility (Jiang et al., 2016). Laminins can act as ligands that bind immune cell membrane receptors (mainly integrins) and initiate integrin-mediated signaling. In LNs, the vascular and lymphatic systems that support T cells trafficking are surrounded by BM which contains two laminin isoforms, laminin 411 and laminin 511 (Simon et al., 2019).

Stiff ECM may act as a physical barrier to T cells infiltration into the tumors and affect the preferential localization of T cells. For example, matrix density and architecture induced the localization and migration of T cells into the tumor stroma rather than into tumor cell nest in both pancreatic ductal adenocarcinoma (PDAC) and lung cancer model due to the impaired ability of T cells to infiltrate from the stroma to cancer cell nests (Salmon et al., 2012; Wolf et al., 2013; Hartmann et al., 2014). In contrast, loose areas of FN and collagen tend to facilitate the motility of T cells (Wolf et al., 2013).

Besides the rigidity, certain ECM components can also play a role in modulating the motility of T cells (He and Baum, 2004; O’Connor et al., 2012). The dense collagen-rich ECM has direct and indirect effects on the infiltration and function of T cells. In a three-dimensional cell culture systems without chemokine gradients, T cells move along the fibrillar collagen network in a process independent of integrin or protease activity. When activated CD8^+^ T cells are encapsulated in collagen hydrogels with distinct fiber alignment, CD8^+^ T cells move along the axis of collagen alignment, and faster and more persistently in aligned collagen fibers than in non-aligned collagen fibers (Pruitt et al., 2020). Additionally, when naive T cells are activated and become T_EFF_ in LNs, T_EFF_ express high levels of CD44 and can bind to HA. CD44 and its engagement with HA skews CD8^+^ T cells toward a terminal effector differentiation state that reduces their ability to form memory cells. As T cell activation proceeds, HA binding increases, and the ability of T cells to roll on HA substrate is improved, which is quite important for the migration and extravasation of T cells (Vogt Sionov and Naor, 1997; Johnson and Jackson, 2021).

### Extracellular Matrix Disturbs the Recognition and Killing of Cancer Cells by T Cells

It is generally believed that CD8^+^ T cells are the main effector cells involved in the killing of cancer cells. Furthermore, killing cancer cells always follows the “recognition” step. In various cancers, collagen fibers are thicker and more closely packed around cancer cell nests than tumor stroma. The stiff ECM can serve as a space barrier surrounding tumor cells and limiting the accessibility by the CD8^+^ T cells, which disturbs the recognition (Stromnes et al., 2015).

Spatial analysis of cancers demonstrates that cancers with excessive ECM deposition are resistant to immune checkpoint inhibition (Mariathasan et al., 2018), which is confirmed by data from multiple lung cancer patients (Peng et al., 2020). Collagen-induced CD8^+^ T cell exhaustion is due to the leukocyte-specific collagen receptor 1 (LAIR1), which suppresses lymphocytic activity through SH2 domain-containing phosphatase 1 (SHP-1) signaling and is expressed on CD8^+^ T cells following integrin beta 2 (CD18) binding to collagen (Lebbink et al., 2007). Reduction in tumor collagen deposition through Lysyl oxidase-like 2 (LOXL2) suppression increases T cell infiltration, diminishes exhausted T cells and abrogates resistance to anti-PD-L1 (Peng et al., 2020). Collagen density reduces the proliferation and tumoricidal activity of tumor-infiltrating T cells. Whole-transcriptome analysis of 3D-cultured T cells revealed that a high-density matrix induces downregulation of cytotoxic activity markers (CD101) and upregulation of regulatory T cell markers (CIP2A). These transcriptional changes have been predicted to involve autocrine TGF-β signaling and they are accompanied by an impaired ability of tumor infiltrating T cells to kill autologous cancer cells (Kuczek et al., 2019).

What’s more, the expression of programmed death-ligand 1 (PD-L1) in tumor cells plays an important role in escaping from the “killing” step (Sun et al., 2018). A stiff substrate enhances the expression of PD-L1 in lung cancer cells through the actin-dependent mechanism, indicating that stiffness as a tumor environment can upregulate the expression of PD-L1 and lead to the escape of the immune system and tumor growth (Miyazawa et al., 2018; Azadi et al., 2019). Researchers are inspired by the effect of tumor-associated ECM on the cancer-immunity cycle stated above. If the negative effect of ECM is eliminated, the anti-tumor immunity would be significantly improved, thus achieving good immunotherapy effect and finally improving overall survival. Therefore, in the next section, we make a summary of strategies to eliminate the negative effect of ECM.

## Strategies to Manipulate the Tumor-Associated Extracellular Matrix

As a vast repository of anticancer targets, ECM has been a hot topic in recent decades (Lorusso et al., 2020), however, the potential for targeting the matrix to promote tumor immunity is less well recognized. The tumor-associated ECM can be therapeutically targeted in a number of ways, including targeting ECM molecules, ECM-remodeling enzymes, altering the structural or physical properties of the matrix, or regulating the fibroblast function as an indirect method to alter ECM deposition (Gordon-Weeks and Yuzhalin, 2020). In the following, we highlight a number of major therapeutic candidates for theregulation of ECM related to cancer immunity.

### Strategies to Directly Target Extracellular Matrix Components

As aforesaid, the components of ECM can act as endogenous mediators of cancer-associated inflammation, which emerges a novel idea that immunosuppression can be improved by targeting them. For instance, some studies demonstrates that the rationality of the modulation of PG’s activities could be a novel approach in the field of cancer immunotherapy. The administration of versikine, a VCAN fragment of bioactive damage-associated molecular pattern, facilitates immune sensing of myeloma tumors and enhances T-cell-activating immunotherapies (Hope et al., 2016). The non-glycanated mouse or human endocan polypeptide (previously known as endothelial cell specific molecule-1, ESM-1) restrains tumor growth by increasing leukocyte infiltration *in vivo* and enhancing innate immunity response (Yassine et al., 2015). The modulation of Syndecans-1 (SDC1) expression could facilitate immunosurveillance and the resolution of precancerous lesions (Handra-Luca, 2020). It has been proved that BGN can bridge innate immunity with adaptive immunity through TLR2/4 downstream signaling because it has the ability to regulate the activities of neutrophils, macrophages, T lymphocytes, B lymphocytes, and DCs (Moreth et al., 2012). Conjugation immune checkpoint antibodies to the heparin binding domain (HBD) of placental growth factor-2 (PIGF-2_123__–__144_) shows exceptionally high affinity for multiple ECM proteins. Peri-tumoral injection of PIGF-2_123__–__144_-anti-PD-L1 leads to higher retention within tumor tissue. More than the high efficacy, it reduces systemic toxicity in the B16F10 melanoma model (Ishihara et al., 2017).

Accumulation of excessive HA can lead to the increase of interstitial pressure and impair the perfusion and chemotherapy delivery of tumors (Jacobetz et al., 2013). In preclinical studies, pegvorhyaluronidase alfa (PEGPH20), a pegylated recombinant human hyaluronidase, has been proved to successfully degrade HA in tumors and remodel the tumor stroma, thus improving perfusion and drug delivery. Therefore, the recent phase II HALO-202 clinical trial (NCT 01839487) showed that treatment with PEGPH20 plus gemcitabine and Nab-paclitaxel significantly improved progression-free survival (PFS) in patients with previously untreated metastatic PDAC (Hingorani et al., 2018). However, the phase III trial suggested that the addition of PEGPH20 to gemcitabine and Nab-paclitaxel increased the ORR but did not improve OS, which did not support further development of PEGPH20 in metastatic PDAC (Van Cutsem et al., 2020). The depletion of HA by hyaluronidase not only improves chemotherapy delivery, but it may also improve the success of immunotherapy, which has been demonstrated by animal models. Moreover, hyaluronidase (HAase) is employed to increase tumor tissues permeability by breaking down the HA in the ECM of tumors to enhance the tumor infiltration of the nanovaccine-generated tumor-specific T cells (Guan et al., 2018). In the presence of hyaluronidase, the delivery of both nano-vaccines and therapeutic monoclonal antibodies has been enhanced (Blair et al., 2019; Clift et al., 2019).

Similarly, collagen I targeting of cetuximab, an anti-EGFR monoclonal antibody, shows promising therapeutic efficacy in the A431 epidermoid cancer model (Liang et al., 2016). Cetuximab can retain a longer period in the collagen-rich tumors without loss of potency (Magdeldin et al., 2014). Collagen affinity also can be used to mediate targeted immunotherapy antibodies, for example, cancer-collagen-targeting immunoconjugate therapy (Katsumata et al., 2019). Fusion of the collagen-binding protein lumican and cytokines increases the efficacy of systemic immunotherapies in a melanoma model. ICIs and interleukin-2 (IL-2) by conjugation (for antibodies) or recombinant fusion (for cytokine) to the von Willebrand factor A3 domain (a collagen-binding domain) eradicated tumors and exhibited obvious safety and efficacy in a breast cancer model (Ishihara et al., 2019).

### Strategies to Indirectly Target Extracellular Matrix Components

In addition to directly targeting or exploiting ECM components, other approaches also focus on targeting the upstream or downstream cellular response of the altered ECM in tumors.

Collagen, the most abundant component of ECM, is mainly secreted by fibroblasts (Pankova et al., 2016). The treatment of collagen in the current study is mainly to target the function of cancer-associated fibroblasts (CAFs) (Liu T. et al., 2019). Fibroblast depletion has primarily focused on the targeting of fibroblast populations with positive fibroblast activation protein (FAP), that improves tumor-associated ECM in lung cancer and pancreatic cancer models (Lo et al., 2015). In murine melanoma model, ablation of FAP-expressing CAFs induced a reduction in immunosuppressive myeloid cells (Zhang and Ertl, 2016). However, a phase II trial of FAP inhibition using the small molecule inhibitor Talabostat failed to demonstrate clinical efficacy for colorectal cancer (Narra et al., 2007). It’s should be noted that targeting CAFs may cause the progression of cancer in the setting of pancreatic adenocarcinoma (Ozdemir et al., 2015). The depletion of α-smooth muscle actin (α-SMA)-positive myofibroblasts reveals the potential for the dissemination and metastasis of diseases (Ding et al., 2014), which emphasizes the importance of understanding the heterogeneity of fibroblasts both within and between cancer subtypes, and is necessary before the targeting of fibroblasts in human tumors. It’s single cell sequencing that probably holds the key to this understanding and enables to identify fibroblast subsets linked to abnormal tumor immunity (Kieffer et al., 2020).

In addition to the depletion of CAFs, other approaches focus on targeting the downstream cellular response to influence ECM. Up to date, the hot targets are the major matrix binding proteins, the integrins and their downstream signaling mechanisms. The use of small molecule kinase inhibitors targeting signaling pathways regulated by ECM such as focal adhesion kinase (FAK) (Jiang et al., 2016) and Rho-associated protein kinase (ROCK) (Johan and Samuel, 2019) has been proved to be effective in targeting the ECM accompanying desmoplasia in cancer.

Based on these preclinical data, several clinical studies have been launched (Table 1). According to https://clinicaltrials.gov/, some clinical studies on targeted ECM have been withdrawn or terminated due to various reasons. However, with the deepening understanding of ECM, people are exploring strategies of transforming the ECM into a strong anti-tumor immunity, and preclinical study and clinical research will continue.

**TABLE 1:** Examples of clinical trials combining agents targeting the tumor ECM with immunotherapy.

**Target**	**Agent**	**Cancer**	**Plus immunotherapy**	**NIH number**	**Trial phase**	**Status/conclusion**
FAK	Defactinib	Pancreatic Cancer	Pembrolizumab	NCT03727880	2	Recruiting
	Defactinib	Advanced solid tumors	Pembrolizumab	NCT02758587	1 and 2	Recruiting
	Defactinib	Advanced solid tumors	Pembrolizumab	NCT02546531	1	Completed
	Defactinib	Pleural mesothelioma	Pembrolizumab	NCT04201145	1	Withdrawn
	Defactinib	Epithelial ovarian cancer	Avelumab	NCT02943317	1	Withdrawn
Collagen	Iosartan	Localized pancreatic cancer	Nivolumab	NCT03563248	2	Recruiting
	Cetuximab	Squamous cell carcinoma of the head and neck	Monalizumab	NCT04590963	3	Recruiting
	Cetuximab	Recurrent/metastatic squamous cell carcinoma of the head and neck	Pembrolizumab	NCT03082534	2	Recruiting
Hyaluronan	PEGPH20	NSCLC and gastric cancer	Pembrolizumab	NCT02563548	1	Completed
	PEGPH20	Metastatic pancreatic cancer	Pembrolizumab	NCT04058964	2	withdrawn
	PEGPH20	HA high metastatic PDAC	Pembrolizumab	NCT03634332	2	Unknown
	PEGPH20	Advanced intrahepatic and extrahepatic cholangiocarcinoma and gallbladder adenocarcinoma	Atezolizumab	NCT03267940	1	Terminated
	PEGPH20	Resectable PDAC	Atezolizumab	NCT03979066	2	Terminated
	PEGPH20	Chemotherapy resistant pancreatic cancer	Avelumab	NCT03481920	1	Terminated
FAP	Talabostat	Advanced and recurrent solid cancer	Pembrolizumab	NCT04171219	2	Recruiting
DDR2	Dasatinib	Advanced non-small cell lung cancer	Nivolumab	NCT02750514	2	Terminated
	Dasatinib	Gastrointestinal stromal tumors or other sarcomas	Ipilimumab	NCT01643278	1	Completed

*FAK, focal adhesion kinase, FAP, fibroblast activation protein, DDR2, discoidin domain receptors 2.*

## Conclusion

Growing evidence suggests that tumor immunity and the response to immunotherapy are affected by many factors besides the antigenicity and/or mutational burden of cancer. This wide array of parameters includes the competency of the host immune system and the composition of the tumor-associated ECM.

A great number of studies on the regulation of ECM in cancer immunity are emerging, but there are still some puzzles. For example, what are the differences in ECM remodeling between immune cold tumors and immune hot tumors? What are the differences in the ECM components between those two populations? So far, we have not yet found more detailed and direct updated literature in this field. However, based on the studies of the effect of ECM on the proliferation and infiltration of T cells, we would propose that collagens and PGs will be the main different components of ECM between “cold” and “hot” tumors. Next, we may conduct a study to compare the differences of ECM between these two populations.

Our review has summarized that the components of ECM play a critical role in regulating each step of the cancer-immunity cycle, which also highlights the potential of targeting tumor-associated ECM to improve cancer immunotherapy. According to the common compositional alterations of ECM (collagen and Hyaluronan) and data from some published studies, PEGPH20, targeting Hyaluronan, and Cetuximab targeting collagen may have the potentiality to be explored, although targeting ECM has not yet yielded satisfactory results as an adjuvant immunotherapy. It is important to note that the compositional and structural complexity of the ECM as well as significant intra-tumoral heterogeneity are still yet to be fully understood, which may limit the targeting of ECM. Fortunately, technological advances such as multiplexed-immunohistochemistry, tissue decellularization techniques, single cell sequencing and mass-spectrometry are beginning to address these issues above. In the future, strategies to manipulate the tumor-associated ECM are expected to produce novel approaches to optimize the therapeutic strategies of novel immunotherapies and prolong the overall survival of cancer.

## Author Contributions

YH, TL, and SD analyzed the data and wrote the manuscript. ZX provided helpful discussion. LW and FL reviewed the manuscript. All authors reviewed the manuscript.

## Conflict of Interest

The authors declare that the research was conducted in the absence of any commercial or financial relationships that could be construed as a potential conflict of interest.

## Publisher’s Note

All claims expressed in this article are solely those of the authors and do not necessarily represent those of their affiliated organizations, or those of the publisher, the editors and the reviewers. Any product that may be evaluated in this article, or claim that may be made by its manufacturer, is not guaranteed or endorsed by the publisher.

## References

[B1] Abbott M. Ustoyev Y. (2019). Cancer and the immune system: the history and background of immunotherapy. *Semin. Oncol. Nurs.* 35:150923. 10.1016/j.soncn.2019.08.002 31526550

[B2] Alaniz L. Garcia M. Rizzo M. Piccioni F. Mazzolini G. (2009). Altered hyaluronan biosynthesis and cancer progression: an immunological perspective. *Mini. Rev. Med. Chem.* 9 1538–1546. 10.2174/138955709790361485 20205635

[B3] Andersson-Sjoland A. Hallgren O. Rolandsson S. Weitoft M. Tykesson E. Larsson-Callerfelt A. K. (2015). Versican in inflammation and tissue remodeling: the impact on lung disorders. *Glycobiology* 25 243–251. 10.1093/glycob/cwu120 25371494 PMC4310351

[B4] Arneth B. (2019). Tumor microenvironment. *Medicina* 56:15. 10.3390/medicina56010015 31906017 PMC7023392

[B5] Aumailley M. (2013). The laminin family. *Cell Adh. Migr.* 7 48–55. 10.4161/cam.22826 23263632 PMC3544786

[B6] Azadi S. Aboulkheyr Es H. Razavi Bazaz S. Thiery J. P. Asadnia M. Ebrahimi Warkiani M. (2019). Upregulation of PD-L1 expression in breast cancer cells through the formation of 3D multicellular cancer aggregates under different chemical and mechanical conditions. *Biochim. Biophys. Acta Mol. Cell Res.* 1866 118526. 10.1016/j.bbamcr.2019.118526 31398408

[B7] Baxevanis C. N. Perez S. A. Papamichail M. (2009). Cancer immunotherapy. *Crit. Rev. Clin. Lab. Sci.* 46 167–189. 10.1080/10408360902937809 19650714

[B8] Bindea G. Mlecnik B. Tosolini M. Kirilovsky A. Waldner M. Obenauf A. C. (2013). Spatiotemporal dynamics of intratumoral immune cells reveal the immune landscape in human cancer. *Immunity* 39 782–795. 10.1016/j.immuni.2013.10.003 24138885

[B9] Blair A. B. Kim V. M. Muth S. T. Saung M. T. Lokker N. Blouw B. (2019). Dissecting the stromal signaling and regulation of myeloid cells and memory effector T cells in pancreatic cancer. *Clin. Cancer Res.* 25 5351–5363. 10.1158/1078-0432.CCR-18-4192 31186314 PMC6726532

[B10] Blander J. M. Longman R. S. Iliev I. D. Sonnenberg G. F. Artis D. (2017). Regulation of inflammation by microbiota interactions with the host. *Nat. Immunol.* 18 851–860. 10.1038/ni.3780 28722709 PMC5800875

[B11] Bonaventura P. Shekarian T. Alcazer V. Valladeau-Guilemond J. Valsesia-Wittmann S. Amigorena S. (2019). Cold tumors: a therapeutic challenge for immunotherapy. *Front. Immunol.* 10:168. 10.3389/fimmu.2019.00168 30800125 PMC6376112

[B12] Bousso P. Robey E. (2003). Dynamics of CD8+ T cell priming by dendritic cells in intact lymph nodes. *Nat. Immunol.* 4 579–585. 10.1038/ni928 12730692

[B13] Bredfeldt J. S. Liu Y. Conklin M. W. Keely P. J. Mackie T. R. Eliceiri K. W. (2014). Automated quantification of aligned collagen for human breast carcinoma prognosis. *J. Pathol. Informat.* 5:28. 10.4103/2153-3539.139707 25250186 PMC4168643

[B14] Brown N. H. (2011). Extracellular matrix in development: insights from mechanisms conserved between invertebrates and vertebrates. *Cold Spring Harb. Perspect. Biol.* 3:a005082. 10.1101/cshperspect.a005082 21917993 PMC3225944

[B15] Bruckner-Tuderman L. von der Mark K. Pihlajaniemi T. Unsicker K. (2010). Cell interactions with the extracellular matrix. *Cell Tissue Res.* 339 1–5. 10.1007/s00441-009-0891-x 19902257

[B16] Burke K. Tang P. Brown E. (2013). Second harmonic generation reveals matrix alterations during breast tumor progression. *J. Biomed. Opt.* 18:31106. 10.1117/1.Jbo.18.3.031106 23172133 PMC3595714

[B17] Caldwell R. W. Rodriguez P. C. Toque H. A. Narayanan S. P. Caldwell R. B. (2018). Arginase: a multifaceted enzyme important in health and disease. *Physiol. Rev.* 98 641–665. 10.1152/physrev.00037.2016 29412048 PMC5966718

[B18] Caon I. Bartolini B. Parnigoni A. Carava E. Moretto P. Viola M. (2020). Revisiting the hallmarks of cancer: the role of hyaluronan. *Semin. Cancer Biol.* 62 9–19. 10.1016/j.semcancer.2019.07.007 31319162

[B19] Chakravarthy A. Khan L. Bensler N. P. Bose P. De Carvalho D. D. (2018). TGF-beta-associated extracellular matrix genes link cancer-associated fibroblasts to immune evasion and immunotherapy failure. *Nat. Commun.* 9:4692. 10.1038/s41467-018-06654-8 30410077 PMC6224529

[B20] Chang J. Chaudhuri O. (2019). Beyond proteases: basement membrane mechanics and cancer invasion. *J. Cell Biol.* 218 2456–2469. 10.1083/jcb.201903066 31315943 PMC6683740

[B21] Chen D. S. Mellman I. (2013). Oncology meets immunology: the cancer-immunity cycle. *Immunity* 39 1–10. 10.1016/j.immuni.2013.07.012 23890059

[B22] Chen D. S. Mellman I. (2017). Elements of cancer immunity and the cancer-immune set point. *Nature* 541 321–330. 10.1038/nature21349 28102259

[B23] Chen S. Y. Lin J. S. Yang B. C. (2014). Modulation of tumor cell stiffness and migration by type IV collagen through direct activation of integrin signaling pathway. *Arch. Biochem. Biophys.* 555 1–8. 10.1016/j.abb.2014.05.004 24823860

[B24] Chimen M. Apta B. H. McGettrick H. M. (2017). Introduction: T cell trafficking in inflammation and immunity. *Methods Mol. Biol.* 1591 73–84. 10.1007/978-1-4939-6931-9_6 28349476

[B25] Choi S. K. Kim H. S. Jin T. Moon W. K. (2017). LOXL4 knockdown enhances tumor growth and lung metastasis through collagen-dependent extracellular matrix changes in triple-negative breast cancer. *Oncotarget* 8 11977–11989. 10.18632/oncotarget.14450 28060764 PMC5355319

[B26] Clift R. Souratha J. Garrovillo S. A. Zimmerman S. Blouw B. (2019). Remodeling the tumor microenvironment sensitizes breast tumors to anti-programmed death-ligand 1 immunotherapy. *Cancer Res.* 79 4149–4159. 10.1158/0008-5472.CAN-18-3060 31248966

[B27] Coelho N. M. McCulloch C. A. (2018). Mechanical signaling through the discoidin domain receptor 1 plays a central role in tissue fibrosis. *Cell Adh. Migr.* 12 348–362. 10.1080/19336918.2018.1448353 29513135 PMC6363045

[B28] Conklin M. W. Eickhoff J. C. Riching K. M. Pehlke C. A. Eliceiri K. W. Provenzano P. P. (2011). Aligned collagen is a prognostic signature for survival in human breast carcinoma. *Am. J. Pathol.* 178 1221–1232. 10.1016/j.ajpath.2010.11.076 21356373 PMC3070581

[B29] Constantino J. Gomes C. Falcao A. Neves B. M. Cruz M. T. (2017). Dendritic cell-based immunotherapy: a basic review and recent advances. *Immunol. Res.* 65 798–810. 10.1007/s12026-017-8931-1 28660480

[B30] Cooper J. Giancotti F. G. (2019). Integrin signaling in cancer: mechanotransduction, stemness, epithelial plasticity, and therapeutic resistance. *Cancer Cell* 35 347–367. 10.1016/j.ccell.2019.01.007 30889378 PMC6684107

[B31] Cox T. R. Erler J. T. (2014). Molecular pathways: connecting fibrosis and solid tumor metastasis. *Clin. Cancer Res.* 20 3637–3643. 10.1158/1078-0432.CCR-13-1059 25028505

[B32] D’Agostino A. Stellavato A. Busico T. Papa A. Tirino V. Papaccio G. (2015). In vitro analysis of the effects on wound healing of high- and low-molecular weight chains of hyaluronan and their hybrid H-HA/L-HA complexes. *BMC Cell Biol.* 16:19. 10.1186/s12860-015-0064-6 26163378 PMC4499215

[B33] D’Angelo S. P. Shoushtari A. N. Keohan M. L. Dickson M. A. Gounder M. M. Chi P. (2017). Combined KIT and CTLA-4 blockade in patients with refractory GIST and other advanced sarcomas: a phase Ib study of dasatinib plus Ipilimumab. *Clin. Cancer Res.* 23 2972–2980. 10.1158/1078-0432.Ccr-16-2349 28007774 PMC5486863

[B34] Darragh L. B. Oweida A. J. Karam S. D. (2018). Overcoming resistance to combination radiation-immunotherapy: a focus on contributing pathways within the tumor microenvironment. *Front. Immunol.* 9:3154. 10.3389/fimmu.2018.03154 30766539 PMC6366147

[B35] Ding L. Zhang Z. Shang D. Cheng J. Yuan H. Wu Y. (2014). alpha-Smooth muscle actin-positive myofibroblasts, in association with epithelial-mesenchymal transition and lymphogenesis, is a critical prognostic parameter in patients with oral tongue squamous cell carcinoma. *J. Oral Pathol. Med.* 43 335–343. 10.1111/jop.12143 24313357

[B36] Drifka C. R. Loeffler A. G. Mathewson K. Mehta G. Keikhosravi A. Liu Y. (2016b). Comparison of picrosirius red staining with second harmonic generation imaging for the quantification of clinically relevant collagen fiber features in histopathology samples. *J. Histochem. Cytochem.* 64 519–529. 10.1369/0022155416659249 27449741 PMC5006137

[B37] Drifka C. R. Loeffler A. G. Mathewson K. Keikhosravi A. Eickhoff J. C. Liu Y. (2016a). Highly aligned stromal collagen is a negative prognostic factor following pancreatic ductal adenocarcinoma resection. *Oncotarget* 7 76197–76213. 10.18632/oncotarget.12772 27776346 PMC5342807

[B38] Du Y. Liu H. He Y. Liu Y. Yang C. Zhou M. (2013). The interaction between LYVE-1 with hyaluronan on the cell surface may play a role in the diversity of adhesion to cancer cells. *PLoS One* 8:e63463. 10.1371/journal.pone.0063463 23717428 PMC3661576

[B39] Dupont S. (2016). Role of YAP/TAZ in cell-matrix adhesion-mediated signalling and mechanotransduction. *Exp. Cell Res.* 343 42–53. 10.1016/j.yexcr.2015.10.034 26524510

[B40] Eble J. A. Niland S. (2019). The extracellular matrix in tumor progression and metastasis. *Clin. Exp. Metastasis* 36 171–198. 10.1007/s10585-019-09966-1 30972526

[B41] El Ghazal R. Yin X. Johns S. C. Swanson L. Macal M. Ghosh P. (2016). Glycan sulfation modulates dendritic cell biology and tumor growth. *Neoplasia* 18 294–306. 10.1016/j.neo.2016.04.004 27237321 PMC4887599

[B42] Erdogan B. Webb D. J. (2017). Cancer-associated fibroblasts modulate growth factor signaling and extracellular matrix remodeling to regulate tumor metastasis. *Biochem. Soc. Trans.* 45 229–236. 10.1042/bst20160387 28202677 PMC5371349

[B43] Fei D. Meng X. Yu W. Yang S. Song N. Cao Y. (2018). Fibronectin (FN) cooperated with TLR2/TLR4 receptor to promote innate immune responses of macrophages via binding to integrin beta1. *Virulence* 9 1588–1600. 10.1080/21505594.2018.1528841 30272511 PMC7000207

[B44] Fletcher A. L. Acton S. E. Knoblich K. (2015). Lymph node fibroblastic reticular cells in health and disease. *Nat. Rev. Immunol.* 15 350–361. 10.1038/nri3846 25998961 PMC5152733

[B45] Fujiwara-Tani R. Sasaki T. Fujii K. Luo Y. Mori T. Kishi S. (2020). Diabetes mellitus is associated with liver metastasis of colorectal cancer through production of biglycan-rich cancer stroma. *Oncotarget* 11 2982–2994. 10.18632/oncotarget.27674 32821344 PMC7415403

[B46] Fukumura D. Kloepper J. Amoozgar Z. Duda D. G. Jain R. K. (2018). Enhancing cancer immunotherapy using antiangiogenics: opportunities and challenges. *Nat. Rev. Clin. Oncol.* 15 325–340. 10.1038/nrclinonc.2018.29 29508855 PMC5921900

[B47] Gadiya M. Chakraborty G. (2018). Signaling by discoidin domain receptor 1 in cancer metastasis. *Cell Adh. Migr.* 12 315–323. 10.1080/19336918.2018.1520556 30187813 PMC6363035

[B48] Gajewski T. F. (2015). The next hurdle in cancer immunotherapy: overcoming the non-T-cell-inflamed tumor microenvironment. *Semin. Oncol.* 42 663–671. 10.1053/j.seminoncol.2015.05.011 26320069 PMC4555998

[B49] Galluzzi L. Chan T. A. Kroemer G. Wolchok J. D. Lopez-Soto A. (2018). The hallmarks of successful anticancer immunotherapy. *Sci. Transl. Med.* 10:eaat7807. 10.1126/scitranslmed.aat7807 30232229

[B50] Gao S. Yang D. Fang Y. Lin X. Jin X. Wang Q. (2019). Engineering nanoparticles for targeted remodeling of the tumor microenvironment to improve cancer immunotherapy. *Theranostics.* 9 126–151. 10.7150/thno.29431 30662558 PMC6332787

[B51] Gao X. Qiao X. Xing X. Huang J. Qian J. Wang Y. (2020). Matrix stiffness-upregulated MICRORNA-17-5p attenuates the intervention effects of metformin on HCC invasion and metastasis by targeting the PTEN/PI3K/Akt pathway. *Front. Oncol.* 10:1563. 10.3389/fonc.2020.01563 32974191 PMC7466473

[B52] Gardner A. Ruffell B. (2016). Dendritic cells and cancer immunity. *Trends Immunol.* 37 855–865. 10.1016/j.it.2016.09.006 27793569 PMC5135568

[B53] Garg A. D. More S. Rufo N. Mece O. Sassano M. L. Agostinis P. (2017). Trial watch: immunogenic cell death induction by anticancer chemotherapeutics. *Oncoimmunology* 6:e1386829. 10.1080/2162402X.2017.1386829 29209573 PMC5706600

[B54] Gkretsi V. Stylianopoulos T. (2018). Cell adhesion and matrix stiffness: coordinating cancer cell invasion and metastasis. *Front. Oncol.* 8:145. 10.3389/fonc.2018.00145 29780748 PMC5945811

[B55] Gordon-Weeks A. Lim S. Y. Yuzhalin A. Lucotti S. Vermeer J. A. F. Jones K. (2019). Tumour-Derived Laminin alpha5 (LAMA5) promotes colorectal liver metastasis growth, branching angiogenesis and notch pathway inhibition. *Cancers* 11:630. 10.3390/cancers11050630 31064120 PMC6562694

[B56] Gordon-Weeks A. Yuzhalin A. E. (2020). Cancer Extracellular Matrix Proteins Regulate Tumour Immunity. *Cancers* 12 3331. 10.3390/cancers12113331 33187209 PMC7696558

[B57] Gretz J. E. Norbury C. C. Anderson A. O. Proudfoot A. E. Shaw S. (2000). Lymph-borne chemokines and other low molecular weight molecules reach high endothelial venules via specialized conduits while a functional barrier limits access to the lymphocyte microenvironments in lymph node cortex. *J. Exp. Med.* 192 1425–1440. 10.1084/jem.192.10.1425 11085745 PMC2193184

[B58] Groom J. R. (2015). Moving to the suburbs: T-cell positioning within lymph nodes during activation and memory. *Immunol. Cell Biol.* 93 330–336. 10.1038/icb.2015.29 25753266

[B59] Grzelczyk W. L. Szemraj J. Jozefowicz-Korczynska M. (2016). The matrix metalloproteinase in larynx cancer. *Postepy Hig. Med. Dosw.* 70 1190–1197. 28026822

[B60] Guan X. Chen J. Hu Y. Lin L. Sun P. Tian H. (2018). Highly enhanced cancer immunotherapy by combining nanovaccine with hyaluronidase. *Biomaterials* 171 198–206. 10.1016/j.biomaterials.2018.04.039 29698869

[B61] Gubin M. M. Artyomov M. N. Mardis E. R. Schreiber R. D. (2015). Tumor neoantigens: building a framework for personalized cancer immunotherapy. *J. Clin. Invest.* 125 3413–3421. 10.1172/JCI80008 26258412 PMC4588307

[B62] Halfter W. Oertle P. Monnier C. A. Camenzind L. Reyes-Lua M. Hu H. (2015). New concepts in basement membrane biology. *FEBS J.* 282 4466–4479. 10.1111/febs.13495 26299746

[B63] Han W. Chen S. Yuan W. Fan Q. Tian J. Wang X. (2016). Oriented collagen fibers direct tumor cell intravasation. *Proc. Natl. Acad. Sci. U.S.A.* 113 11208–11213. 10.1073/pnas.1610347113 27663743 PMC5056065

[B64] Hanahan D. Weinberg R. A. (2011). Hallmarks of cancer: the next generation. *Cell* 144 646–674. 10.1016/j.cell.2011.02.013 21376230

[B65] Handra-Luca A. (2020). Syndecan-1 in the tumor microenvironment. *Adv. Exp. Med. Biol.* 1272 39–53. 10.1007/978-3-030-48457-6_3 32845501

[B66] Haraida S. Nerlich A. G. Wiest I. Schleicher E. Löhrs U. (1996). Distribution of basement membrane components in normal adipose tissue and in benign and malignant tumors of lipomatous origin. *Mod. Pathol.* 9 137–144. 8657720

[B67] Hartmann N. Giese N. A. Giese T. Poschke I. Offringa R. Werner J. (2014). Prevailing role of contact guidance in intrastromal T-cell trapping in human pancreatic cancer. *Clin. Cancer Res.* 20 3422–3433. 10.1158/1078-0432.Ccr-13-2972 24763614

[B68] He J. Baum L. G. (2004). Presentation of galectin-1 by extracellular matrix triggers T cell death. *J. Biol. Chem.* 279 4705–4712. 10.1074/jbc.M311183200 14617626

[B69] He X. Lee B. Jiang Y. (2016). Cell-ECM interactions in tumor invasion. *Adv. Exp. Med. Biol.* 936 73–91. 10.1007/978-3-319-42023-3_4 27739043

[B70] Hingorani S. R. Zheng L. Bullock A. J. Seery T. E. Harris W. P. Sigal D. S. (2018). HALO 202: randomized phase II study of PEGPH20 plus nab-paclitaxel/gemcitabine versus nab-paclitaxel/gemcitabine in patients with untreated, metastatic pancreatic ductal adenocarcinoma. *J. Clin. Oncol.* 36 359–366. 10.1200/JCO.2017.74.9564 29232172

[B71] Hope C. Foulcer S. Jagodinsky J. Chen S. X. Jensen J. L. Patel S. (2016). Immunoregulatory roles of versican proteolysis in the myeloma microenvironment. *Blood* 128 680–685. 10.1182/blood-2016-03-705780 27259980 PMC4974200

[B72] Horvath L. Thienpont B. Zhao L. Wolf D. Pircher A. (2020). Overcoming immunotherapy resistance in non-small cell lung cancer (NSCLC) - novel approaches and future outlook. *Mol. Cancer* 19:141. 10.1186/s12943-020-01260-z 32917214 PMC7488475

[B73] Huang Y. L. Liang C. Y. Ritz D. Coelho R. Septiadi D. Estermann M. (2020). Collagen-rich omentum is a premetastatic niche for integrin α2-mediated peritoneal metastasis. *Elife* 9:e59442. 10.7554/eLife.59442 33026975 PMC7541088

[B74] Humphrey J. D. Dufresne E. R. Schwartz M. A. (2014). Mechanotransduction and extracellular matrix homeostasis. *Nat. Rev. Mol. Cell Biol.* 15 802–812. 10.1038/nrm3896 25355505 PMC4513363

[B75] Huse M. (2017). Mechanical forces in the immune system. *Nat. Rev. Immunol.* 17 679–690. 10.1038/nri.2017.74 28757604 PMC6312705

[B76] Hwang H. J. Oh M. S. Lee D. W. Kuh H. J. (2019). Multiplex quantitative analysis of stroma-mediated cancer cell invasion, matrix remodeling, and drug response in a 3D co-culture model of pancreatic tumor spheroids and stellate cells. *J. Exp. Clin. Cancer Res.* 38:258. 10.1186/s13046-019-1225-9 31200779 PMC6567511

[B77] Iozzo R. V. Schaefer L. (2015). Proteoglycan form and function: a comprehensive nomenclature of proteoglycans. *Matrix Biol.* 42 11–55. 10.1016/j.matbio.2015.02.003 25701227 PMC4859157

[B78] Ishihara J. Fukunaga K. Ishihara A. Larsson H. M. Potin L. Hosseinchi P. (2017). Matrix-binding checkpoint immunotherapies enhance antitumor efficacy and reduce adverse events. *Sci. Transl. Med.* 9:eaan0401. 10.1126/scitranslmed.aan0401 29118259

[B79] Ishihara J. Ishihara A. Sasaki K. Lee S. S. Williford J. M. Yasui M. (2019). Targeted antibody and cytokine cancer immunotherapies through collagen affinity. *Sci. Transl. Med.* 11:eaau3259. 10.1126/scitranslmed.aau3259 30971453 PMC6541444

[B80] Jacobetz M. A. Chan D. S. Neesse A. Bapiro T. E. Cook N. Frese K. K. (2013). Hyaluronan impairs vascular function and drug delivery in a mouse model of pancreatic cancer. *Gut* 62 112–120. 10.1136/gutjnl-2012-302529 22466618 PMC3551211

[B81] Jakubzick C. V. Randolph G. J. Henson P. M. (2017). Monocyte differentiation and antigen-presenting functions. *Nat. Rev. Immunol.* 17 349–362. 10.1038/nri.2017.28 28436425

[B82] Jiang H. Hegde S. Knolhoff B. L. Zhu Y. Herndon J. M. Meyer M. A. (2016). Targeting focal adhesion kinase renders pancreatic cancers responsive to checkpoint immunotherapy. *Nat. Med.* 22 851–860. 10.1038/nm.4123 27376576 PMC4935930

[B83] Jie H. B. Gildener-Leapman N. Li J. Srivastava R. M. Gibson S. P. Whiteside T. L. (2013). Intratumoral regulatory T cells upregulate immunosuppressive molecules in head and neck cancer patients. *Br. J. Cancer.* 109 2629–2635. 10.1038/bjc.2013.645 24169351 PMC3833228

[B84] Johan M. Z. Samuel M. S. (2019). Rho-ROCK signaling regulates tumor-microenvironment interactions. *Biochem. Soc. Trans.* 47 101–108. 10.1042/BST20180334 30559270

[B85] Johnson L. A. Jackson D. G. (2021). Hyaluronan and its receptors: key mediators of immune cell entry and trafficking in the lymphatic system. *Cells* 10:2061. 10.3390/cells10082061 34440831 PMC8393520

[B86] Jurmeister P. von Laffert M. Johrens K. (2020). Dissecting the spatial heterogeneity of different immune cell subsets in non-small cell lung cancer. *Pathol. Res. Pract.* 216:152904. 10.1016/j.prp.2020.152904 32143905

[B87] Kang H. Wu Q. Sun A. Liu X. Fan Y. Deng X. (2018). Cancer cell glycocalyx and its significance in cancer progression. *Int. J. Mol. Sci.* 19:2484. 10.3390/ijms19092484 30135409 PMC6163906

[B88] Katakai T. Hara T. Sugai M. Gonda H. Shimizu A. (2004). Lymph node fibroblastic reticular cells construct the stromal reticulum via contact with lymphocytes. *J. Exp. Med.* 200 783–795. 10.1084/jem.20040254 15381731 PMC2211971

[B89] Katsumata K. Ishihara J. Mansurov A. Ishihara A. Raczy M. M. Yuba E. (2019). Targeting inflammatory sites through collagen affinity enhances the therapeutic efficacy of anti-inflammatory antibodies. *Sci. Adv.* 5:eaay1971. 10.1126/sciadv.aay1971 31723606 PMC6834392

[B90] Kaylan K. B. Gentile S. D. Milling L. E. Bhinge K. N. Kosari F. Underhill G. H. (2016). Mapping lung tumor cell drug responses as a function of matrix context and genotype using cell microarrays. *Integr. Biol.* 8 1221–1231. 10.1039/c6ib00179c 27796394

[B91] Kieffer Y. Hocine H. R. Gentric G. Pelon F. Bernard C. Bourachot B. (2020). Single-cell analysis reveals fibroblast clusters linked to immunotherapy resistance in cancer. *Cancer Discov.* 10 1330–1351. 10.1158/2159-8290.CD-19-1384 32434947

[B92] Kim S. Takahashi H. Lin W. W. Descargues P. Grivennikov S. Kim Y. (2009). Carcinoma-produced factors activate myeloid cells through TLR2 to stimulate metastasis. *Nature* 457 102–106. 10.1038/nature07623 19122641 PMC2746432

[B93] Kodaira Y. Nair S. K. Wrenshall L. E. Gilboa E. Platt J. L. (2000). Phenotypic and functional maturation of dendritic cells mediated by heparan sulfate. *J. Immunol.* 165 1599–1604. 10.4049/jimmunol.165.3.1599 10903769

[B94] Kong X. (2020). Discovery of new immune checkpoints: family grows up. *Adv. Exp. Med. Biol.* 1248 61–82. 10.1007/978-981-15-3266-5_4 32185707

[B95] Kroemer G. Galluzzi L. Kepp O. Zitvogel L. (2013). Immunogenic cell death in cancer therapy. *Annu. Rev. Immunol.* 31 51–72. 10.1146/annurev-immunol-032712-100008 23157435

[B96] Kuczek D. E. Larsen A. M. H. Thorseth M. L. Carretta M. Kalvisa A. Siersbaek M. S. (2019). Collagen density regulates the activity of tumor-infiltrating T cells. *J. Immunother. Cancer* 7:68. 10.1186/s40425-019-0556-6 30867051 PMC6417085

[B97] Kumar A. V. Katakam S. K. Urbanowitz A. K. Gotte M. (2015). Heparan sulphate as a regulator of leukocyte recruitment in inflammation. *Curr. Protein Pept. Sci.* 16 77–86. 10.2174/1573402111666150213165054 25692849

[B98] Laczko R. Csiszar K. (2020). Lysyl oxidase (LOX): functional contributions to signaling pathways. *Biomolecules* 10:1093. 10.3390/biom10081093 32708046 PMC7465975

[B99] Larsen A. M. H. Kuczek D. E. Kalvisa A. Siersbæk M. S. Thorseth M. L. Johansen A. Z. (2020). Collagen density modulates the immunosuppressive functions of macrophages. *J. Immunol.* 205 1461–1472. 10.4049/jimmunol.1900789 32839214

[B100] Lebbink R. J. de Ruiter T. Kaptijn G. J. Bihan D. G. Jansen C. A. Lenting P. J. (2007). Mouse leukocyte-associated Ig-like receptor-1 (mLAIR-1) functions as an inhibitory collagen-binding receptor on immune cells. *Int. Immunol.* 19 1011–1019. 10.1093/intimm/dxm071 17702987

[B101] Leenman E. E. Mukhina M. S. Girshovich M. M. Kanaev S. V. Konoplev S. N. Pozharisskii K. M. (2010). [The place of dendritic cells in the microenviroment in Hodgkin’s lymphoma]. *Arkh. Patol.* 72 3–7. 20698307

[B102] Li B. Chan H. L. Chen P. (2019). Immune checkpoint inhibitors: basics and challenges. *Curr. Med. Chem.* 26 3009–3025. 10.2174/0929867324666170804143706 28782469

[B103] Li H. X. Zheng J. H. Fan H. X. Li H. P. Gao Z. X. Chen D. (2013). Expression of αvβ6 integrin and collagen fibre in oral squamous cell carcinoma: association with clinical outcomes and prognostic implications. *J. Oral Pathol. Med.* 42 547–556. 10.1111/jop.12044 23331428

[B104] Li L. Shirkey M. W. Zhang T. Xiong Y. Piao W. Saxena V. (2020). The lymph node stromal laminin α5 shapes alloimmunity. *J. Clin. Invest.* 130 2602–2619. 10.1172/jci135099 32017712 PMC7190966

[B105] Liang H. Li X. Wang B. Chen B. Zhao Y. Sun J. (2016). A collagen-binding EGFR antibody fragment targeting tumors with a collagen-rich extracellular matrix. *Sci. Rep.* 6:18205. 10.1038/srep18205 26883295 PMC4756367

[B106] Lin T. C. Yang C. H. Cheng L. H. Chang W. T. Lin Y. R. Cheng H. C. (2019). Fibronectin in Cancer: Friend or Foe. *Cells* 9 27. 10.3390/cells9010027 31861892 PMC7016990

[B107] Litwiniuk M. Krejner A. Speyrer M. S. Gauto A. R. Grzela T. (2016). Hyaluronic acid in inflammation and tissue regeneration. *Wounds* 28 78–88. 26978861

[B108] Liu M. Tolg C. Turley E. (2019). Dissecting the dual nature of hyaluronan in the tumor microenvironment. *Front. Immunol.* 10:947. 10.3389/fimmu.2019.00947 31134064 PMC6522846

[B109] Liu T. Han C. Wang S. Fang P. Ma Z. Xu L. (2019). Cancer-associated fibroblasts: an emerging target of anti-cancer immunotherapy. *J. Hematol. Oncol.* 12:86. 10.1186/s13045-019-0770-1 31462327 PMC6714445

[B110] Liu Y. Li W. Li X. Tai Y. Lü Q. Yang N. (2014). Expression and significance of biglycan in endometrial cancer. *Arch. Gynecol. Obstetr.* 289 649–655. 10.1007/s00404-013-3017-3 24013431

[B111] Lo A. Wang L. S. Scholler J. Monslow J. Avery D. Newick K. (2015). Tumor-promoting desmoplasia is disrupted by depleting FAP-expressing stromal cells. *Cancer Res.* 75 2800–2810. 10.1158/0008-5472.CAN-14-3041 25979873 PMC4506263

[B112] Löffek S. Franzke C. W. Helfrich I. (2016). Tension in cancer. *Int. J. Mol. Sci.* 17:1910. 10.3390/ijms17111910 27854331 PMC5133907

[B113] Lorusso G. Ruegg C. Kuonen F. (2020). Targeting the extra-cellular matrix-tumor cell crosstalk for anti-cancer therapy: emerging alternatives to integrin inhibitors. *Front. Oncol.* 10:1231. 10.3389/fonc.2020.01231 32793493 PMC7387567

[B114] Lu P. Weaver V. M. Werb Z. (2012). The extracellular matrix: a dynamic niche in cancer progression. *J. Cell Biol.* 196 395–406. 10.1083/jcb.201102147 22351925 PMC3283993

[B115] Madsen D. H. Bugge T. H. (2015). The source of matrix-degrading enzymes in human cancer: problems of research reproducibility and possible solutions. *J. Cell Biol.* 209 195–198. 10.1083/jcb.201501034 25918222 PMC4411277

[B116] Magdeldin T. Lopez-Davila V. Villemant C. Cameron G. Drake R. Cheema U. (2014). The efficacy of cetuximab in a tissue-engineered three-dimensional in vitro model of colorectal cancer. *J. Tissue Eng.* 5:2041731414544183. 10.1177/2041731414544183 25383169 PMC4221936

[B117] Mariathasan S. Turley S. J. Nickles D. Castiglioni A. Yuen K. Wang Y. (2018). TGFbeta attenuates tumour response to PD-L1 blockade by contributing to exclusion of T cells. *Nature* 554 544–548. 10.1038/nature25501 29443960 PMC6028240

[B118] Martinez V. G. Pankova V. Krasny L. Singh T. Makris S. White I. J. (2019). Fibroblastic reticular cells control conduit matrix deposition during lymph node expansion. *Cell Rep.* 29:e5. 10.1016/j.celrep.2019.10.103 31775047 PMC6899512

[B119] Mason J. A. Hagel K. R. Hawk M. A. Schafer Z. T. (2017). Metabolism during ECM detachment: achilles heel of cancer cells? *Trends Cancer* 3 475–481. 10.1016/j.trecan.2017.04.009 28718402

[B120] Masopust D. Schenkel J. M. (2013). The integration of T cell migration, differentiation and function. *Nat. Rev. Immunol.* 13 309–320. 10.1038/nri3442 23598650

[B121] Mattheolabakis G. Milane L. Singh A. Amiji M. M. (2015). Hyaluronic acid targeting of CD44 for cancer therapy: from receptor biology to nanomedicine. *J. Drug Target* 23 605–618. 10.3109/1061186X.2015.1052072 26453158

[B122] Mecham R. P. (2012). Overview of extracellular matrix. *Curr. Protoc. Cell Biol.* 10:10. 10.1002/0471143030.cb1001s57 23208544

[B123] Mempel T. R. Henrickson S. E. Von Andrian U. H. (2004). T-cell priming by dendritic cells in lymph nodes occurs in three distinct phases. *Nature* 427 154–159. 10.1038/nature02238 14712275

[B124] Misra S. Hascall V. C. Markwald R. R. Ghatak S. (2015). Interactions between Hyaluronan and Its Receptors (CD44, RHAMM) regulate the activities of inflammation and cancer. *Front. Immunol.* 6:201. 10.3389/fimmu.2015.00201 25999946 PMC4422082

[B125] Miyazawa A. Ito S. Asano S. Tanaka I. Sato M. Kondo M. (2018). Regulation of PD-L1 expression by matrix stiffness in lung cancer cells. *Biochem. Biophys. Res. Commun.* 495 2344–2349. 10.1016/j.bbrc.2017.12.115 29274784

[B126] Mohan V. Das A. Sagi I. (2020). Emerging roles of ECM remodeling processes in cancer. *Semin. Cancer Biol.* 62 192–200. 10.1016/j.semcancer.2019.09.004 31518697

[B127] Monslow J. Govindaraju P. Puré E. (2015). Hyaluronan - a functional and structural sweet spot in the tissue microenvironment. *Front. Immunol.* 6:231. 10.3389/fimmu.2015.00231 26029216 PMC4432798

[B128] Moreth K. Iozzo R. V. Schaefer L. (2012). Small leucine-rich proteoglycans orchestrate receptor crosstalk during inflammation. *Cell Cycle* 11 2084–2091. 10.4161/cc.20316 22580469 PMC3368860

[B129] Mueller B. M. Schraufstatter I. U. Goncharova V. Povaliy T. DiScipio R. Khaldoyanidi S. K. (2010). Hyaluronan inhibits postchemotherapy tumor regrowth in a colon carcinoma xenograft model. *Mol. Cancer Therapeut.* 9 3024–3032. 10.1158/1535-7163.Mct-10-0529 20833754 PMC2978790

[B130] Naci D. Vuori K. Aoudjit F. (2015). Alpha2beta1 integrin in cancer development and chemoresistance. *Semin. Cancer Biol.* 35 145–153. 10.1016/j.semcancer.2015.08.004 26297892

[B131] Najafi M. Farhood B. Mortezaee K. (2019). Extracellular matrix (ECM) stiffness and degradation as cancer drivers. *J. Cell Biochem.* 120 2782–2790. 10.1002/jcb.27681 30321449

[B132] Nakamura K. Smyth M. J. (2017). Targeting cancer-related inflammation in the era of immunotherapy. *Immunol. Cell Biol.* 95 325–332. 10.1038/icb.2016.126 27999432

[B133] Narra K. Mullins S. R. Lee H. O. Strzemkowski-Brun B. Magalong K. Christiansen V. J. (2007). Phase II trial of single agent Val-boroPro (Talabostat) inhibiting fibroblast activation protein in patients with metastatic colorectal cancer. *Cancer Biol. Ther.* 6 1691–1699. 10.4161/cbt.6.11.4874 18032930

[B134] Navab R. Strumpf D. To C. Pasko E. Kim K. S. Park C. J. (2016). Integrin α11β1 regulates cancer stromal stiffness and promotes tumorigenicity and metastasis in non-small cell lung cancer. *Oncogene* 35 1899–1908. 10.1038/onc.2015.254 26148229 PMC4833874

[B135] Neill T. Schaefer L. Iozzo R. V. (2015). Decoding the matrix: instructive roles of proteoglycan receptors. *Biochemistry* 54 4583–4598. 10.1021/acs.biochem.5b00653 26177309 PMC4859759

[B136] Ngambenjawong C. Gustafson H. H. Pun S. H. (2017). Progress in tumor-associated macrophage (TAM)-targeted therapeutics. *Adv. Drug Deliv. Rev.* 114 206–221. 10.1016/j.addr.2017.04.010 28449873 PMC5581987

[B137] Nikitovic D. Papoutsidakis A. Karamanos N. K. Tzanakakis G. N. (2014). Lumican affects tumor cell functions, tumor-ECM interactions, angiogenesis and inflammatory response. *Matrix Biol.* 35 206–214. 10.1016/j.matbio.2013.09.003 24060754

[B138] Nikitovic D. Tzardi M. Berdiaki A. Tsatsakis A. Tzanakakis G. N. (2015). Cancer microenvironment and inflammation: role of hyaluronan. *Front. Immunol.* 6:169. 10.3389/fimmu.2015.00169 25926834 PMC4396412

[B139] Nissen N. I. Karsdal M. Willumsen N. (2019). Collagens and cancer associated fibroblasts in the reactive stroma and its relation to cancer biology. *J. Exp. Clin. Cancer Res.* 38:115. 10.1186/s13046-019-1110-6 30841909 PMC6404286

[B140] Nolan J. Mahdi A. F. Dunne C. P. Kiely P. A. (2020). Collagen and fibronectin promote an aggressive cancer phenotype in breast cancer cells but drive autonomous gene expression patterns. *Gene* 761:145024. 10.1016/j.gene.2020.145024 32755659

[B141] Nolte M. Margadant C. (2020). Controlling immunity and inflammation through integrin-dependent regulation of TGF-β. *Trends Cell Biol.* 30 49–59. 10.1016/j.tcb.2019.10.002 31744661

[B142] O’Connor R. S. Hao X. Shen K. Bashour K. Akimova T. Hancock W. W. (2012). Substrate rigidity regulates human T cell activation and proliferation. *J. Immunol.* 189 1330–1339. 10.4049/jimmunol.1102757 22732590 PMC3401283

[B143] Ohno S. Tachibana M. Fujii T. Ueda S. Kubota H. Nagasue N. (2002). Role of stromal collagen in immunomodulation and prognosis of advanced gastric carcinoma. *Int. J. Cancer* 97 770–774. 10.1002/ijc.10144 11857352

[B144] Orgel J. Madhurapantula R. S. (2019). A structural prospective for collagen receptors such as DDR and their binding of the collagen fibril. *Biochim. Biophys. Acta Mol. Cell Res.* 1866 118478. 10.1016/j.bbamcr.2019.04.008 31004686

[B145] Ozdemir B. C. Pentcheva-Hoang T. Carstens J. L. Zheng X. Wu C. C. Simpson T. R. (2015). Depletion of carcinoma-associated fibroblasts and fibrosis induces immunosuppression and accelerates pancreas cancer with reduced survival. *Cancer Cell* 28 831–833. 10.1016/j.ccell.2015.11.002 28843279

[B146] Pankova D. Chen Y. Terajima M. Schliekelman M. J. Baird B. N. Fahrenholtz M. (2016). Cancer-associated fibroblasts induce a collagen cross-link switch in tumor stroma. *Mol. Cancer Res.* 14 287–295. 10.1158/1541-7786.MCR-15-0307 26631572 PMC4794404

[B147] Pao W. Ooi C. H. Birzele F. Ruefli-Brasse A. Cannarile M. A. Reis B. (2018). Tissue-specific immunoregulation: a call for better understanding of the “Immunostat” in the context of cancer. *Cancer Discov.* 8 395–402. 10.1158/2159-8290.CD-17-1320 29545369

[B148] Papadas A. Arauz G. Cicala A. Wiesner J. Asimakopoulos F. (2020). Versican and versican-matrikines in cancer progression, inflammation, and immunity. *J. Histochem. Cytochem.* 68 871–885. 10.1369/0022155420937098 32623942 PMC7711242

[B149] Paszek M. J. Zahir N. Johnson K. R. Lakins J. N. Rozenberg G. I. Gefen A. (2005). Tensional homeostasis and the malignant phenotype. *Cancer Cell* 8 241–254. 10.1016/j.ccr.2005.08.010 16169468

[B150] Peng D. H. Rodriguez B. L. Diao L. Chen L. Wang J. Byers L. A. (2020). Collagen promotes anti-PD-1/PD-L1 resistance in cancer through LAIR1-dependent CD8(+) T cell exhaustion. *Nat. Commun.* 11:4520. 10.1038/s41467-020-18298-8 32908154 PMC7481212

[B151] Phillippi B. Singh M. Loftus T. Smith H. Muccioli M. Wright J. (2020). Effect of laminin environments and tumor factors on the biology of myeloid dendritic cells. *Immunobiology* 225:151854. 10.1016/j.imbio.2019.10.003 31753553

[B152] Pickup M. W. Mouw J. K. Weaver V. M. (2014). The extracellular matrix modulates the hallmarks of cancer. *EMBO Rep.* 15 1243–1253. 10.15252/embr.201439246 25381661 PMC4264927

[B153] Piersma B. Hayward M. K. Weaver V. M. (2020). Fibrosis and cancer: a strained relationship. *Biochim. Biophys. Acta Rev. Cancer* 1873:188356. 10.1016/j.bbcan.2020.188356 32147542 PMC7733542

[B154] Piperigkou Z. Mohr B. Karamanos N. Gotte M. (2016). Shed proteoglycans in tumor stroma. *Cell Tissue Res.* 365 643–655. 10.1007/s00441-016-2452-4 27365088

[B155] Pruitt H. C. Lewis D. Ciccaglione M. Connor S. Smith Q. Hickey J. W. (2020). Collagen fiber structure guides 3D motility of cytotoxic T lymphocytes. *Matrix Biol.* 8 147–159. 10.1016/j.matbio.2019.02.003 30776427 PMC6697628

[B156] Ribas A. (2015). Adaptive immune resistance: how cancer protects from immune attack. *Cancer Discov.* 5 915–919. 10.1158/2159-8290.CD-15-0563 26272491 PMC4560619

[B157] Ribatti D. (2017). The concept of immune surveillance against tumors. the first theories. *Oncotarget* 8 7175–7180. 10.18632/oncotarget.12739 27764780 PMC5351698

[B158] Roozendaal R. Mebius R. E. Kraal G. (2008). The conduit system of the lymph node. *Int. Immunol.* 20 1483–1487. 10.1093/intimm/dxn110 18824503

[B159] Rotman J. Koster B. D. Jordanova E. S. Heeren A. M. de Gruijl T. D. (2019). Unlocking the therapeutic potential of primary tumor-draining lymph nodes. *Cancer Immunol. Immunother.* 68 1681–1688. 10.1007/s00262-019-02330-y 30944963 PMC6805797

[B160] Saitakis M. Dogniaux S. Goudot C. Bufi N. Asnacios S. Maurin M. (2017). Different TCR-induced T lymphocyte responses are potentiated by stiffness with variable sensitivity. *Elife* 6:e23190. 10.7554/eLife.23190 28594327 PMC5464771

[B161] Salmon H. Franciszkiewicz K. Damotte D. Dieu-Nosjean M. C. Validire P. Trautmann A. (2012). Matrix architecture defines the preferential localization and migration of T cells into the stroma of human lung tumors. *J. Clin. Invest.* 122 899–910. 10.1172/jci45817 22293174 PMC3287213

[B162] Salzer B. Schueller C. M. Zajc C. U. Peters T. Schoeber M. A. Kovacic B. (2020). Engineering AvidCARs for combinatorial antigen recognition and reversible control of CAR function. *Nat. Commun.* 11:4166. 10.1038/s41467-020-17970-3 32820173 PMC7441178

[B163] Schaefer L. Tredup C. Gubbiotti M. A. Iozzo R. V. (2017). Proteoglycan neofunctions: regulation of inflammation and autophagy in cancer biology. *FEBS J.* 284 10–26. 10.1111/febs.13963 27860287 PMC5226885

[B164] Schedin P. Keely P. J. (2011). Mammary gland ECM remodeling, stiffness, and mechanosignaling in normal development and tumor progression. *Cold Spring Harb. Perspect. Biol.* 3:a003228. 10.1101/cshperspect.a003228 20980442 PMC3003460

[B165] Simon T. Li L. Wagner C. Zhang T. Saxena V. Brinkman C. C. (2019). Differential regulation of T-cell immunity and tolerance by stromal laminin expressed in the lymph node. *Transplantation* 103 2075–2089. 10.1097/tp.0000000000002774 31343575 PMC6768765

[B166] Singh N. Baby D. Rajguru J. P. Patil P. B. Thakkannavar S. S. Pujari V. B. (2019). Inflammation and cancer. *Ann. Afr. Med.* 18 121–126. 10.4103/aam.aam_56_18 31417011 PMC6704802

[B167] Skandalis S. S. Karalis T. T. Chatzopoulos A. Karamanos N. K. (2019). Hyaluronan-CD44 axis orchestrates cancer stem cell functions. *Cell Signal.* 63:109377. 10.1016/j.cellsig.2019.109377 31362044

[B168] Sorushanova A. Delgado L. M. Wu Z. Shologu N. Kshirsagar A. Raghunath R. (2019). The collagen suprafamily: from biosynthesis to advanced biomaterial development. *Adv. Mater.* 31:e1801651. 10.1002/adma.201801651 30126066

[B169] Sprague L. Muccioli M. Pate M. Meles E. McGinty J. Nandigam H. (2011). The interplay between surfaces and soluble factors define the immunologic and angiogenic properties of myeloid dendritic cells. *BMC Immunol.* 12:35. 10.1186/1471-2172-12-35 21645356 PMC3124423

[B170] Stromnes I. M. Schmitt T. M. Hulbert A. Brockenbrough J. S. Nguyen H. Cuevas C. (2015). T cells engineered against a native antigen can surmount immunologic and physical barriers to treat pancreatic ductal adenocarcinoma. *Cancer Cell* 28 638–652. 10.1016/j.ccell.2015.09.022 26525103 PMC4724422

[B171] Stylianopoulos T. Munn L. L. Jain R. K. (2018). Reengineering the physical microenvironment of tumors to improve drug delivery and efficacy: from mathematical modeling to bench to bedside. *Trends Cancer* 4 292–319. 10.1016/j.trecan.2018.02.005 29606314 PMC5930008

[B172] Subbarayan K. Leisz S. Wickenhauser C. Bethmann D. Massa C. Steven A. (2018). Biglycan-mediated upregulation of MHC class I expression in HER-2/neu-transformed cells. *Oncoimmunology* 7:e1373233. 10.1080/2162402x.2017.1373233 29632715 PMC5889282

[B173] Sun C. Mezzadra R. Schumacher T. N. (2018). Regulation and function of the PD-L1 Checkpoint. *Immunity* 48 434–452. 10.1016/j.immuni.2018.03.014 29562194 PMC7116507

[B174] Tabuso M. Adya R. Stark R. Gopalakrishnan K. Tsang Y. W. James S. (2021). Fibrotic phenotype of peritumour mesenteric adipose tissue in human colon cancer: a potential hallmark of metastatic properties. *Int. J. Mol. Sci.* 22:2430. 10.3390/ijms22052430 33670920 PMC7957668

[B175] Termeer C. Benedix F. Sleeman J. Fieber C. Voith U. Ahrens T. (2002). Oligosaccharides of Hyaluronan activate dendritic cells via toll-like receptor 4. *J. Exp. Med.* 195 99–111. 10.1084/jem.20001858 11781369 PMC2196009

[B176] Theocharis A. D. Skandalis S. S. Gialeli C. Karamanos N. K. (2016). Extracellular matrix structure. *Adv. Drug Deliv. Rev.* 97 4–27. 10.1016/j.addr.2015.11.001 26562801

[B177] Tian X. Azpurua J. Hine C. Vaidya A. Myakishev-Rempel M. Ablaeva J. (2013). High-molecular-mass hyaluronan mediates the cancer resistance of the naked mole rat. *Nature* 499 346–349. 10.1038/nature12234 23783513 PMC3720720

[B178] Törrönen K. Nikunen K. Kärnä R. Tammi M. Tammi R. Rilla K. (2014). Tissue distribution and subcellular localization of hyaluronan synthase isoenzymes. *Histochem. Cell Biol.* 141 17–31. 10.1007/s00418-013-1143-4 24057227

[B179] Tu M. M. Lee F. Y. F. Jones R. T. Kimball A. K. Saravia E. Graziano R. F. (2019). Targeting DDR2 enhances tumor response to anti-PD-1 immunotherapy. *Sci. Adv.* 5:eaav2437. 10.1126/sciadv.aav2437 30801016 PMC6382401

[B180] Vaday G. G. Franitza S. Schor H. Hecht I. Brill A. Cahalon L. (2001). Combinatorial signals by inflammatory cytokines and chemokines mediate leukocyte interactions with extracellular matrix. *J. Leukoc. Biol.* 69 885–892. 11404372

[B181] Vaday G. G. Lider O. (2000). Extracellular matrix moieties, cytokines, and enzymes: dynamic effects on immune cell behavior and inflammation. *J. Leukoc. Biol.* 67 149–159. 10.1002/jlb.67.2.149 10670574

[B182] Van Cutsem E. Tempero M. A. Sigal D. Oh D. Y. Fazio N. Macarulla T. (2020). Randomized phase III trial of pegvorhyaluronidase alfa with nab-paclitaxel plus gemcitabine for patients with hyaluronan-high metastatic pancreatic adenocarcinoma. *J. Clin. Oncol.* 38 3185–3194. 10.1200/JCO.20.00590 32706635 PMC7499614

[B183] Vennin C. Pajic M. Timpson P. (2015). Imaging fibrosis in pancreatic cancer using second harmonic generation. *Pancreatology* 15 200–201. 10.1016/j.pan.2015.02.004 26020072

[B184] Vogt Sionov R. Naor D. (1997). Hyaluronan-independent lodgment of CD44+ lymphoma cells in lymphoid organs. *Int. J. Cancer* 71 462–469. 10.1002/(sici)1097-0215(19970502)71:3<462::aid-ijc26<3.0.co;2-g 9139885

[B185] Walker C. Mojares E. Del Rio Hernandez A. (2018). Role of extracellular matrix in development and cancer progression. *Int. J. Mol. Sci.* 19:3028. 10.3390/ijms19103028 30287763 PMC6213383

[B186] Wang B. Li G. X. Zhang S. G. Wang Q. Wen Y. G. Tang H. M. (2011). Biglycan expression correlates with aggressiveness and poor prognosis of gastric cancer. *Exp. Biol. Med.* 236 1247–1253. 10.1258/ebm.2011.011124 21998129

[B187] Wang J. Yang T. Xu J. (2020). Therapeutic development of immune checkpoint inhibitors. *Adv. Exp. Med. Biol.* 1248 619–649. 10.1007/978-981-15-3266-5_23 32185726

[B188] Wang Q. Ju X. Wang J. Fan Y. Ren M. Zhang H. (2018). Immunogenic cell death in anticancer chemotherapy and its impact on clinical studies. *Cancer Lett.* 438 17–23. 10.1016/j.canlet.2018.08.028 30217563

[B189] Wang S. He Z. Wang X. Li H. Liu X. S. (2019). Antigen presentation and tumor immunogenicity in cancer immunotherapy response prediction. *Elife* 8:e49020. 10.7554/eLife.49020 31767055 PMC6879305

[B190] Warren K. J. Iwami D. Harris D. G. Bromberg J. S. Burrell B. E. (2014). Laminins affect T cell trafficking and allograft fate. *J. Clin. Invest.* 124 2204–2218. 10.1172/jci73683 24691446 PMC4001556

[B191] Watanabe H. (2010). [Extracellular matrix–regulation of cancer invasion and metastasis]. *Gan Kagaku Ryoho* 37 2058–2061. 21084803

[B192] Wculek S. K. Cueto F. J. Mujal A. M. Melero I. Krummel M. F. Sancho D. (2020). Dendritic cells in cancer immunology and immunotherapy. *Nat. Rev. Immunol.* 20 7–24. 10.1038/s41577-019-0210-z 31467405

[B193] Wight T. N. (2017). Provisional matrix: a role for versican and hyaluronan. *Matrix Biol.* 6 38–56. 10.1016/j.matbio.2016.12.001 27932299 PMC5438907

[B194] Wight T. N. Kang I. Merrilees M. J. (2014). Versican and the control of inflammation. *Matrix Biol.* 35 152–161. 10.1016/j.matbio.2014.01.015 24513039 PMC4039577

[B195] Winer A. Adams S. Mignatti P. (2018). Matrix metalloproteinase inhibitors in cancer therapy: turning past failures into future successes. *Mol. Cancer Therapeut.* 17 1147–1155. 10.1158/1535-7163.Mct-17-0646 29735645 PMC5984693

[B196] Wolf K. Te Lindert M. Krause M. Alexander S. Te Riet J. Willis A. L. (2013). Physical limits of cell migration: control by ECM space and nuclear deformation and tuning by proteolysis and traction force. *J. Cell Biol.* 201 1069–1084. 10.1083/jcb.201210152 23798731 PMC3691458

[B197] Worbs T. Hammerschmidt S. I. Forster R. (2017). Dendritic cell migration in health and disease. *Nat. Rev. Immunol.* 17 30–48. 10.1038/nri.2016.116 27890914

[B198] Wrenshall L. E. Carlson A. Cerra F. B. Platt J. L. (1994). Modulation of cytolytic T cell responses by heparan sulfate. *Transplantation* 57 1087–1094. 8165708

[B199] Wu X. Cai J. Zuo Z. Li J. (2019). Collagen facilitates the colorectal cancer stemness and metastasis through an integrin/PI3K/AKT/Snail signaling pathway. *Biomed. Pharmacother.* 114:108708. 10.1016/j.biopha.2019.108708 30913493

[B200] Xu S. Xu H. Wang W. Li S. Li H. Li T. (2019). The role of collagen in cancer: from bench to bedside. *J. Transl. Med.* 17:309. 10.1186/s12967-019-2058-1 31521169 PMC6744664

[B201] Yassine H. De Freitas Caires N. Depontieu F. Scherpereel A. Awad A. Tsicopoulos A. (2015). The non glycanated endocan polypeptide slows tumor growth by inducing stromal inflammatory reaction. *Oncotarget* 6 2725–2735. 10.18632/oncotarget.2614 25575808 PMC4413613

[B202] Yue B. (2014). Biology of the extracellular matrix: an overview. *J. Glaucoma* 23 S20–S23. 10.1097/ijg.0000000000000108 25275899 PMC4185430

[B203] Zeltz C. Gullberg D. (2016). The integrin-collagen connection–a glue for tissue repair? *J. Cell Sci.* 129 653–664. 10.1242/jcs.180992 26857815

[B204] Zemek R. M. De Jong E. Chin W. L. Schuster I. S. Fear V. S. Casey T. H. (2019). Sensitization to immune checkpoint blockade through activation of a STAT1/NK axis in the tumor microenvironment. *Sci. Transl. Med.* 11:eaav7816. 10.1126/scitranslmed.aav7816 31316010

[B205] Zeng-Brouwers J. Beckmann J. Nastase M. V. Iozzo R. V. Schaefer L. (2014). De novo expression of circulating biglycan evokes an innate inflammatory tissue response via MyD88/TRIF pathways. *Matrix Biol.* 35 132–142. 10.1016/j.matbio.2013.12.003 24361484 PMC4039567

[B206] Zhang J. Zhao R. Li B. Farrukh A. Hoth M. Qu B. (2021). Micropatterned soft hydrogels to study the interplay of receptors and forces in T cell activation. *Acta Biomater.* 119 234–246. 10.1016/j.actbio.2020.10.028 33099024

[B207] Zhang Y. Ertl H. C. (2016). Depletion of FAP+ cells reduces immunosuppressive cells and improves metabolism and functions CD8+T cells within tumors. *Oncotarget* 7 23282–23299. 10.18632/oncotarget.7818 26943036 PMC5029626

[B208] Zhang Y. Zheng J. (2020). Functions of immune checkpoint molecules beyond immune evasion. *Adv. Exp. Med. Biol.* 1248 201–226. 10.1007/978-981-15-3266-5_9 32185712

